# The riddle of mitochondrial alkaline/neutral invertases: A novel Arabidopsis isoform mainly present in reproductive tissues and involved in root ROS production

**DOI:** 10.1371/journal.pone.0185286

**Published:** 2017-09-25

**Authors:** Marina E. Battaglia, María Victoria Martin, Leandra Lechner, Giselle M. A. Martínez-Noël, Graciela L. Salerno

**Affiliations:** Instituto de Investigaciones en Biodiversidad y Biotecnología (INBIOTEC-CONICET) and Fundación para Investigaciones Biológicas Aplicadas (FIBA), Mar del Plata, Argentina; Institute of Genetics and Developmental Biology Chinese Academy of Sciences, CHINA

## Abstract

Alkaline/neutral invertases (A/N-Inv), glucosidases that irreversibly hydrolyze sucrose into glucose and fructose, play significant roles in plant growth, development, and stress adaptation. They occur as multiple isoforms located in the cytosol or organelles. In *Arabidopsis thaliana*, two mitochondrial *A/N-Inv* genes (*A/N-InvA* and *A/N-InvC*) have already been investigated. In this study, we functionally characterized *A/N-InvH*, a third Arabidopsis gene coding for a mitochondrial-targeted protein. The phenotypic analysis of knockout mutant plants (*invh*) showed a severely reduced shoot growth, while root development was not affected. The emergence of the first floral bud and the opening of the first flower were the most affected stages, presenting a significant delay. *A/N-InvH* transcription is markedly active in reproductive tissues. It is also expressed in the elongation and apical meristem root zones. Our results show that *A/N-InvH* expression is not evident in photosynthetic tissues, despite being of relevance in developmental processes and mitochondrial functional status. NaCl and mannitol treatments increased *A/N-InvH* expression twofold in the columella root cap. Moreover, the absence of *A/N-InvH* prevented ROS formation, not only in *invh* roots of salt- and ABA-treated seedlings but also in *invh* control roots. We hypothesize that this isoform may take part in the ROS/sugar (sucrose or its hydrolysis products) signaling pathway network, involved in reproductive tissue development, cell elongation, and abiotic stress responses.

## Introduction

In addition to their crucial role in respiration, plant mitochondria are also involved in many other central cellular processes to meet the specific demands of photosynthetic organisms. In this respect, they must coordinate gene functions with other organelles [1]. Mitochondrial respiration is an important fate for hexoses, largely derived from sucrose, a major end-product of the photosynthetic process [2]. Although the entrance of cytosolic sugars could occur, either across the mitochondrial outer membrane [3] or via sugar transporters located in the inner membrane, neither sucrose nor glucose nor fructose is likely to accumulate in the mitochondrial matrix [4].

Sucrose occupies a key position in plant life as the major form of transport from photosynthetic (mainly mature leaves) to heterotrophic tissues (such as roots, seeds, flowers, fruits) providing carbon skeletons and energy for biosynthesis. Sucrose also has an important role in response to environmental stress and as a primary messenger in signal transduction. The disaccharide and/or its hydrolysis products (glucose and fructose) can act as important metabolic signals, modulating gene expression and regulating plant growth and development [5–7]. Sucrose is synthesized in the cytosol of the plant cell through the successive action of sucrose-phosphate synthase and sucrose-phosphate phosphatase [8]. However, its utilization by metabolism can be achieved by two distinct enzymatic activities (sucrose synthase and invertase) [9], which, can display different subcellular localizations [7,10]. Sucrose synthase (EC 2.4.1.13), a glucosyltransferase that reversibly catalyzes sucrose conversion to UDP-glucose and fructose, can be localized in the cytosol, bound to the plasmatic membrane or associated with mitochondria. It provides substrate for storage or structural polysaccharides’ biosynthesis (such as starch and cellulose) and for energy generation (ATP) [8,9,11]. On the other hand, invertases irreversibly hydrolyze sucrose into glucose and fructose, which enter the metabolism after phosphorylation by hexokinases or fulfill a signaling role [9]. Invertases play key functions in primary metabolism and plant development [12]. According to their pH optimum, they were formerly classified into acid (pH between 4.5 and 5.0) and alkaline/neutral (A/N-Invs, pH between 6.5 and 8.0) invertases [13,14], which share no sequence homology and have different subcellular localizations in the plant cell. Acid invertases, fructofuranosidases belonging to the glycoside hydrolase family 32, are found in the vacuole or cell wall and have been extensively studied [14,15]. On the contrary, the knowledge on A/N-Invs is rather scant, since they have been barely investigated in recent decades, probably due to their low concentration and labile activity [10]. The availability of complete sequenced genomes (from both cyanobacteria and plants) allowed the execution of thorough investigations on those proteins that showed different isoforms, located either in the cytosol or inside the organelles (mitochondrion, chloroplast, or nucleus). Studies in different plant species indicated that A/N-Invs are involved in carbon distribution, cellular differentiation, tissue development and stress responses [10]. It has recently been revealed, from structural, catalytic and substrate specificity studies on InvA from *Anabaena* sp. PCC 7120 that A/N-Invs belong to a novel family of glucosidases that specifically catalyze the cleavage of the α-1,2-glycosidic bond of sucrose [16].

In recent years, A/N-Inv gene families (containing from 7 to 16 genes) have been identified in several plant species (i.e., *Arabidopsis thaliana*, *Oryza sativa*, *Populus trichocharpa*, *Vitis vinifera*, *Lotus japonicus*, *Malus × domestica*, and *Manihot esculenta* [17–25]. Particularly, of the nine members of the *A/N-Inv* gene family in *A*. *thaliana* [17,18], five codify for cytosolic proteins, one (or two) for a chloroplast-target protein, and three (or two) for mitochondrial isoforms [21,26–28]. Arabidopsis *A/N-InvA* [29] and *A/N-InvC* [30] were functionally characterized by heterologous expression in *E*. *coli* as encoding A/N-Inv proteins of mitochondrial location. This was demonstrated by either transient expression in protoplasts of a chimerical construction (the full-length or the N-terminal region of *A/N-InvA* fused to the GFP encoding gene) [29], or in vivo experiments using GFP translational fusions, in the case of A/N-InvC [30]. In the Arabidopsis genome, there is a third sequence, predicted as coding for a putative plastid/mitochondrial A/N-Inv [10], which has not been investigated so far.

In the present study, we functionally characterized the genomic Arabidopsis sequence corresponding to locus At3g05820 as coding for a mitochondrial protein with A/N-Inv activity, hereinafter named *A/N-InvH*. Phenotype analysis of knockout plants (*invh*) showed a severely reduced shoot growth and time delay in the first stages of flowering. *A/N-InvH* is expressed mainly in reproductive tissues, and its absence impaired ROS production in roots of seedlings not only subjected to salt or osmotic stress but also kept under control conditions. Our results reinforce the relevance of the three mitochondrial A/N-Inv isoforms with significant and different roles in plant development. Our data also point to an increasing complexity in the regulation of plant life and stress responses, through an intricate signaling pathway network, involving sucrose, hexoses, abscisic acid (ABA) and ROS.

## Materials and methods

### Biological material and growth conditions

Seeds from *Arabidopsis thaliana* (wild-type, wt) ecotype Columbia 0 (Col-0) and from a T-DNA insertional mutant SALK_103674.18.70.x [31] were obtained from the Arabidopsis Biological Resource Centre (ABRC, Ohio State University). The mutant (hereinafter referred to as *invh*) was a homozygous knockout line for the locus At3g05820 (S1 Fig). After superficial sterilization, seeds were placed on Murashige-Skoog (MS) agar plates containing 1% sucrose and Mes-KOH buffer (pH 5.7), and maintained at 4°C for three days in the dark to synchronize germination. Then plates were moved to a growth chamber under controlled conditions of light and temperature (16 h light/8 h dark, 22°C). After seven days, seedlings were transferred to pots containing soil mixture (3:1:1 mixture of peat moss-based mix:vermiculite:perlite).

Black bean (*Phaseolus vulgaris*) seeds, obtained from a commercial market (Legumbres Elio, http://legumbreselio.com/, Coronel Dominguez, Santa Fe Province, Argentina) were used for the fusion protein expression in hairy roots. Seeds were superficially sterilized and germinated on wet paper in dark conditions at 28°C [32]. After three days, seedlings were transferred to vermiculite for further infection with *Agrobacterium rhizogenes* strain K599 (a kind gift from Flavio Blanco, IBBM-CONICET-UNLP, Argentina) and cultured as previously described [33].

*Escherichia coli* DH5α and BL21(λDE3):pLysS (Novagen) strains, used for cloning and recombinant protein production, respectively, were routinely grown at 37°C in Luria–Bertani medium supplemented with 30 μg ml^-1^ chloramphenicol and 50 μg ml^-1^ carbenicillin.

*Agrobacterium tumefaciens* GV3101, used for the *A/N-InvH* promoter activity assay in Arabidopsis, was grown in Luria broth medium under agitation for 18 h at 28 C with the addition of gentamycin and rifampicin.

### Phenotypic analysis

To compare wt and *invh* plant growth, a developmental stage-based analysis was conducted following conditions and procedures similar to those reported by Boyes et al. (2001) [34]. Data were collected on a daily basis at the same time using plate-based or soil-based plants from three independent experiments (n = 30 plants). Statistical analyses (ANOVA) were carried out using Prism software.

Germination assays were carried out on MS plates containing 0.05% Mes-KOH (pH 5.7), 1% sucrose and 0.8% agar. Before plating, seeds were surface-sterilized in 96% ethanol for 1 min, and 40% (v/v) sodium hypochlorite plus 0.02% (v/v) Triton X100 for 7 min, and rinsed four to five times with sterile water. After three-days-stratification period at 4°C in the dark, plates were incubated under controlled conditions of temperature and photoperiod (at 22±1°C, 16 h/8 h, light/dark cycle) and germination (defined as radicle emergence from the seed coat) was registered under stereoscopic microscope [35].

### Invertase activity assay

A/N-Inv activity was routinely assayed at 30°C in a 50-μl reaction mixture containing 200 mM Hepes-NaOH (pH 6.5) and 200 mM sucrose. The resulting reducing sugars were quantified as previously reported [36]. The effect of pH on enzyme activity was carried out following Martin et al. (2013) [30]. A/N substrate specificity was tested with sucrose, raffinose, stachyose, melizitose, 1-kestose, trehalose or maltose at 100 mM final concentration in a 50-μl mixture containing 200 mM Hepes-NaOH (pH 6.5) and an aliquot of the recombinant A/N-InvH.

### Bioinformatic analysis for subcellular localization

Subcellular localization was predicted using the following softwares: MitoProt II v1.101, (https://ihg.gsf.de/ihg/mitoprot.html) [37], TargetP1.1 (http://www.cbs.dtu.dk/services/TargetP/) [38], Protein Prowler 1.2 (http://bioinf.scmb.uq.edu.au:8080/pprowler_webapp_1-2) [39], and PSORT for plant sequences (http://www.psort.org/) [40].

### Nucleic acid isolation and manipulation

Plasmids were isolated and modified according to standard protocols [41]. Total DNA extraction from the Arabidopsis lines as well as total RNA isolation and purification were carried out as previously described [30].

### Heterologous expression of *A/N-InvH* in *Escherichia coli*

A 1,749-bp fragment (from the mitochondrial cleavage site at amino-acid residue 44, according to MitoProt II v1.101 prediction [37]) of the *A/N-InvH* gene was PCR-amplified with a specific primer pair [pSETA-InvH-Fw, GGGGTACC CTCGGTTCTCAAGCTGCATC, and pSETA-InvH-Rv CGGAATTCTTAT CGTGAACATTTCTTCCGGC, carrying *Kpn*I and *EcoR*1 restriction sites (underlined nucleotides), respectively]. The amplified fragment was cloned in pGEM™-T Easy Vector System (Promega) and then sub-cloned between the *Kpn*I and *EcoR*1 restriction sites of the expression vector pRSET-A (Invitrogen). The construct was transferred to *E*. *coli* BL21(λDE3)pLysS cells (Novagen) for recombinant protein expression, which was obtained after four-hours induction with 1 mM isopropyl β-D-thiogalactoside at 30°C. His-tagged protein (His_6_::A/N-InvH) was purified by TALON® Co^+2^-affinity resin (Clontech Laboratories, Inc), following the manufacturer’s instructions. After elution with 250 mM EDTA, protein was dialyzed and concentrated for further characterization. Protein determination and A/N-Inv activity was assayed in aliquots of the purified His_6_::A/N-InvH recombinant protein as previously described [30].

### Expression of a fusion protein in *P*. *vulgaris* hairy roots

An *A/N-InvH*::*gfp* fusion was constructed in the pCambia1302 (http://www.cambia.org) vector. A 795-bp fragment from the translational start codon of the *A/N-InvH* encoding region was PCR-amplified from the TAIR clone (DKLAT3G05820) corresponding to At3g05820 locus using the primer pair pC1302-InvH-fw (CCATGGAAATGAATGCCATCACTTTTCTTG) and pC1302-InvH-rv (CAGATCTCGACCTATAGCTGCTTCGCC), which were designed with an adapter for *Nco*I or *Bgl*II restriction enzyme (underlined nucleotides), respectively. The amplified fragment was cloned in pGEM™- T Easy Vector System (Promega). The plasmid was digested with *Nco*I and *Bgl*II restriction enzymes and the resulting DNA fragment was subcloned in the pCambia1302 (http://www.cambia.org) plasmid digested with *Nco*I and *Bgl*II to obtain a fusion to *gfp*. This construction was called pCambia1302-35S::*A/N-InvH*:*gfp*. All constructions were confirmed by sequencing (Macrogen, Korea).

*P*. *vulgaris* composite plants were obtained after infection with *A*. *rhizogenes* transformed with pCambia1302-35S::*A/N-InvH*:*gfp* or pCambia1302 as previously described [32]. Briefly, seeds superficially sterilized with 96% ethanol and 5% (v/v) sodium hypochlorite [42] were germinated on wet paper at 30°C for three days in the dark. Seedlings were transferred to vermiculite and, when cotyledons were fully opened, plants were injected in the inter-cotyledon region with a concentrated suspension of *A*. *rhizogenes* [43]. Hairy roots, developed from the infection site after fifteen days approximately were analyzed with a confocal laser scanning microscope (Nikon Eclipse C1 Plus). Mitochondria were stained with the specific MitoTracker® Red CMXRos probe (Thermo Fisher), following the manufacturers’ protocol. Confocal images were processed and analyzed using ImageJ [44] (NIH ImageJ 1.51i) and Fiji [45].

### *A/N-invH* expression in Arabidopsis stable transgenic lines

A 1.90-kb fragment of the *A/N****-****invH* gene promoter region was PCR amplified from Col-0 genomic DNA using the primer pair SacI_2KbProInvH-Fw (CGAGCTCGGTAACAATGCATTCGACCAGA) and NcoI_2KbProInvH-Rv (CCCATGGAGGCTTTCTTGTGTTCGTTATT), and cloned in pCambia1303 vector. After sequencing the resulting pC1303 *ProinvH*::*gfp* plasmid was transferred into *A*. *tumefaciens* strain GV3101, which was then used to transform Arabidopsis Col-0 plants by floral dip [46]. *ProinvH*::*gfp* transgenic lines were obtained after germination in MS selection medium (MS + hygromycin-B 50 μg ml^-1^).

### Salt, osmotic, oxidative and ABA treatments

Seven-day-old transgenic plants expressing *ProinvH*::*gfp* fusion were incubated in vertical plates with MS medium and 100 μM ABA (abscisic acid), or 100 mM NaCl, or 200 mM mannitol for 24 h. Treatment with 1 mM H_2_O_2_ was performed for 30 min in MS solution. Wild-type plants were used as autofluorescence controls, After treatments, GFP fluorescence was observed in a confocal microscope. Images were collected using the same confocal parameter settings as in control conditions.

### Determination of mitochondrial membrane potential

This analysis was performed with JC-1 (a kind gift from Eduardo Zabaleta, IIB-CONICET, Mar del Plata, Argentina), a lipophilic dye that can selectively enter into the mitochondria. A reversible color change (from green to red) is produced when the membrane potential increases. Seven-day-old wt and *invh* seedlings, grown on vertical plates, were incubated in a 10 μg ml^-1^ JC-1 solution (purchased in Molecular Probes) for 30 min at room temperature, following previous protocols [47,48]. Images were collected using a confocal microscope (Nikon Eclipse C1 Plus) and the intensities of green (excitation/emission wavelength = 485/538 nm) and red (excitation/emission wavelength = 485/590 nm) fluorescence were analyzed for wt and *invh* roots from seven-day-old plants. Images were analyzed using ImageJ software as previously described [49]. A dispersion graph was made with the ratio red to green fluorescence of JC-1 images.

### ROS detection and image analysis

The fluorescent probe 2',7'-dichlorodihydrofluorescein diacetate (H_2_DCFDA) was used for detection studies of reactive oxygen species (ROS). This probe mainly provides a qualitative estimate and localization of general ROS (such as HO-, ROO^-^, ONOO^-^), and, in a minor extent, H_2_O_2_. H_2_DCFDA was prepared as a 10 mM stock solution in DMSO and kept at -20°C. Working solution was a 1:1000 dilution in 20 mM Hepes-NaOH buffer (pH 7.2) according to Distéfano et al. (2017) [50]. Arabidopsis wt and *invh* 7-days-old seedlings were grown on vertical plates and submerged in different solutions (100 mM NaCl, 200 mM mannitol, 100 μM ABA or 1 mM H_2_O_2_) buffered with 20 mM Hepes-NaOH (pH 7.2). After 30 min, plants were transferred to the H_2_DCFDA working solution for 10 min. All root images were acquired with the same exposition time and software setting.

## Results

### *A/N-InvH* gene encodes a functional A/N-Inv

The *A*. *thaliana* genome sequence corresponding to At3g05820 locus (called *A/N-InvH*) was the third putative gene predicted as coding for an organelle-targeted A/N-Inv isoform [17,18,29,30] (S1 and S2 Tables). While the other two genes (*A/N-InvC* and *A/N-InvA*) were functionally characterized as coding for mitochondrial proteins [29,30], *A/N-InvH* was first considered by Vargas et al. (2008) [28] as a sequence coding for a putative plastid protein, expressed exclusively in flowers. To date, *A/N-InvH* has been neither characterized nor studied, probably due to its null or undetectable expression in stems, roots and leaves [28]. This is in line with the comparative analysis performed in the GENEVESTIGATOR browser (www.genevestigator.com) [51] (S2 Fig).

**Fig 1:**
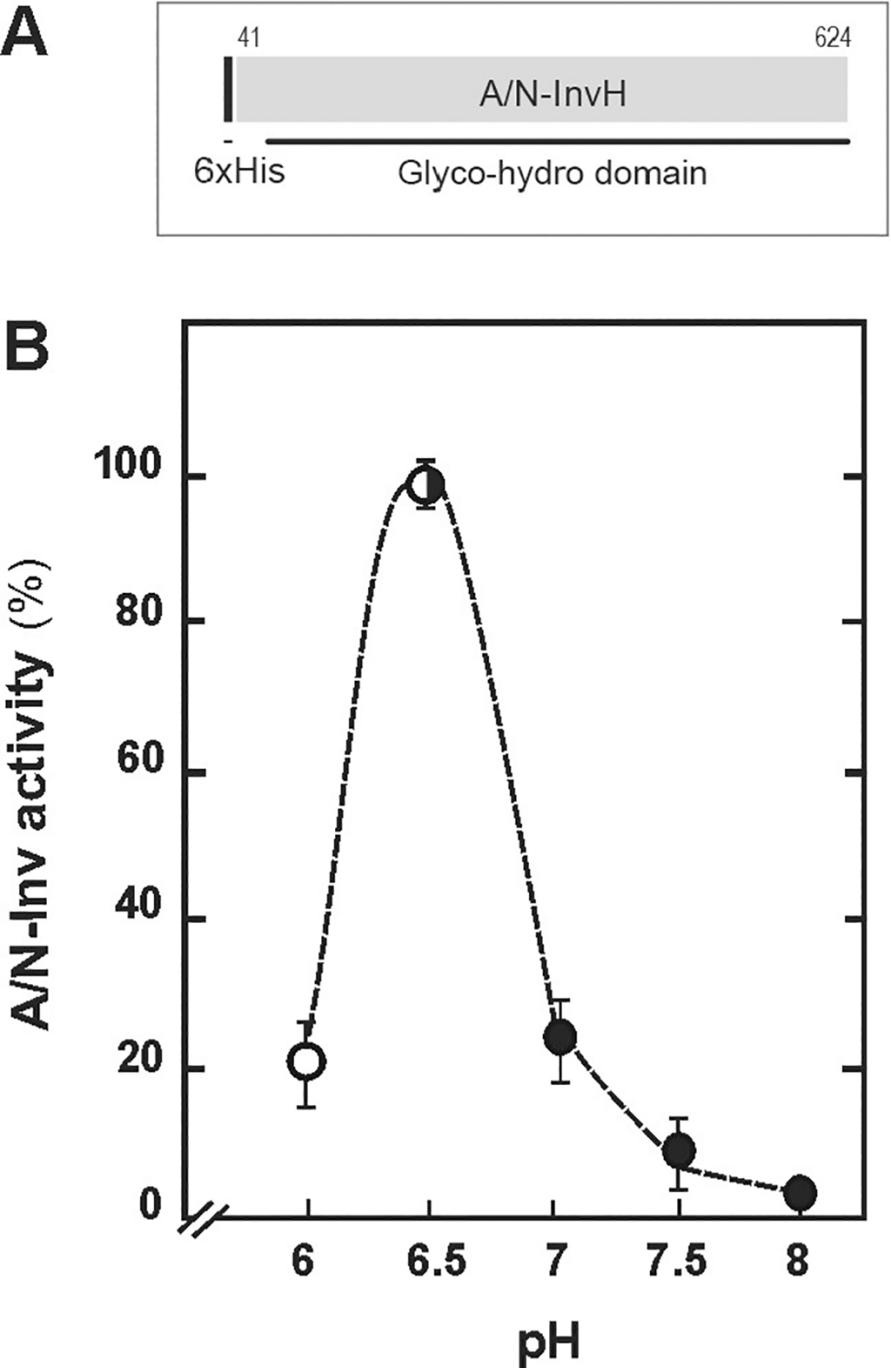
Biochemical characterization of the recombinant A/N-InvH. **(A)** Schematic representation of the recombinant His_6_::A/N-InvH protein. The His-tagged protein lacks the predicted N-terminal transit peptide (1 to 40 amino-acid residues) and contains the glyco-hydro domain (Pfam 12899), characteristic of A/N-Inv proteins. **(B)** A/N-Inv activity of the purified His_6_::A/N-InvH as a function of the pH. To obtain different pHs, potassium phosphate (open circles) or Hepes-NaOH buffer (black circles) were added to the assay mixture.

The *A/N-InvH* sequence encodes a predicted 633-amino-acid protein (~ 72 kDa, UNIPROT: Q84JL5), containing at the N-terminus, a putative mitochondrial transit peptide of 44 or 53 amino-acid residues, according to MitoProt II or PSORT software, respectively (S2 Table). For functional characterization, the *A/N-InvH* sequence was PCR-amplified, cloned and heterologously expressed in *E*. *coli*. The purified His_6_::A/N-InvH protein exhibited specific sucrose hydrolysis activity, which was higher at pH around 6.5 (Fig 1), similar to A/N-InvC [30].

### A/N-InvH is a mitochondrial protein

**Fig 2:**
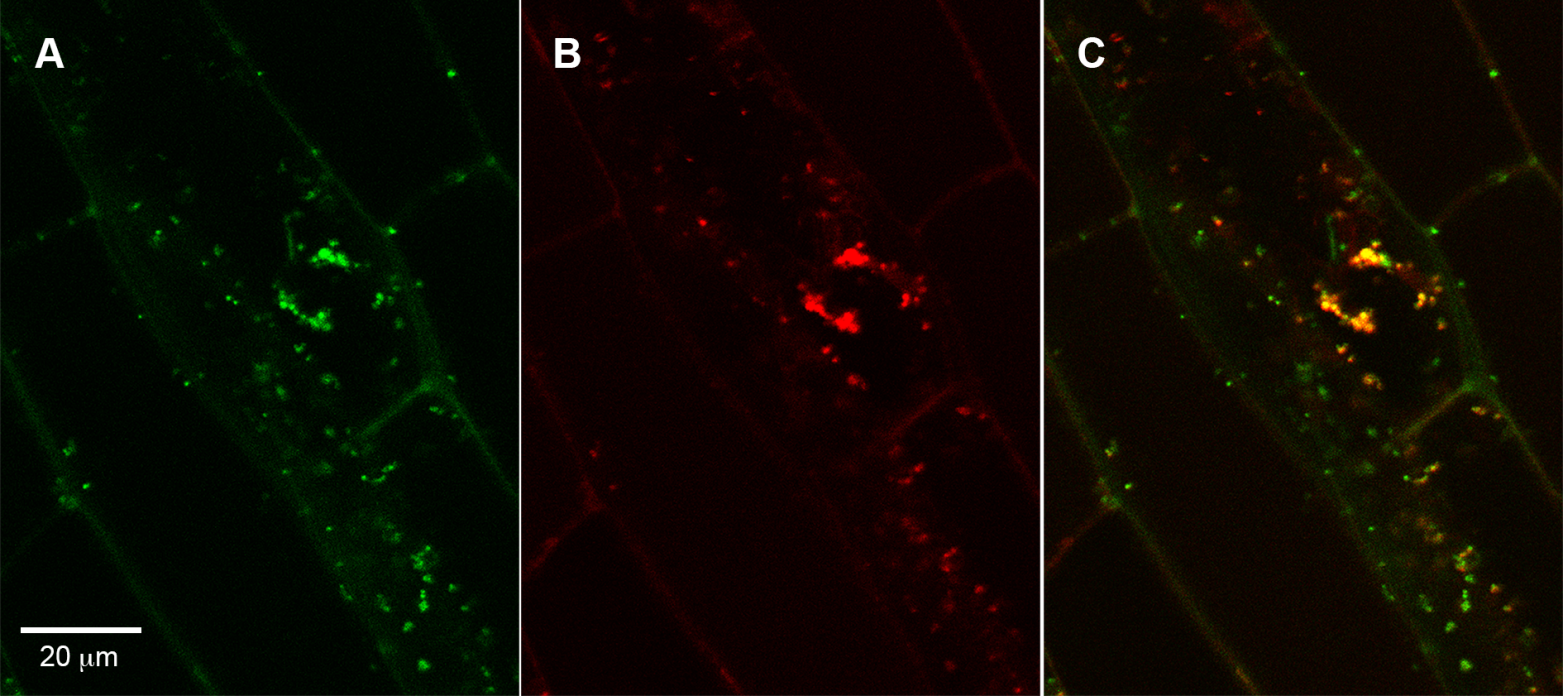
Subcellular localization of the protein product of the *A/N-InvH* gene. A translational fusion of *gfp* and the A/N-InvH encoding sequence was introduced downstream of the CaMV 35S promoter in the plasmid pCambia1302, and the construct was transferred to *A*. *rhizogenes* to generate *P*. *vulgaris* composite plants. After 15 days, hairy roots were analyzed by confocal microscopy. **(A)** GFP fluorescence of the A/N-InvH::GFP fusion protein; **(B)** Visualization of mitochondria with MitoTracker Red; **(C)** Merged image, superimposing the images of GFP and the MitoTracker probe.

To study A/N-InvH subcellular localization, an *A/N-InvH* fragment encoding the first 265 amino-acid residues was in-frame fused upstream of *gfp*, driven by CaMV 35S promoter. The resulting construct was used to transform *P*. *vulgaris* seedlings via *A*. *rhizogenes*. Mitochondrial localization was evaluated after comparing the distribution of the GFP fluorescence and a mitochondrial red-dye specific probe (MitoTracker) in the hairy root cells, using a confocal microscope. As shown in Fig 2, the green fluorescence co-localizes with the red pattern due to the mitochondrion-selective probe. This location was confirmed after analysis of transient transformated *N*. *benthamiana* leaves with an *A/N-InvH*:*gfp* fusion (S3 Fig), where GFP fluorescence was only detected in mitochondria.

### Growth phenotype of *invh* knockout plants

**Fig 3:**
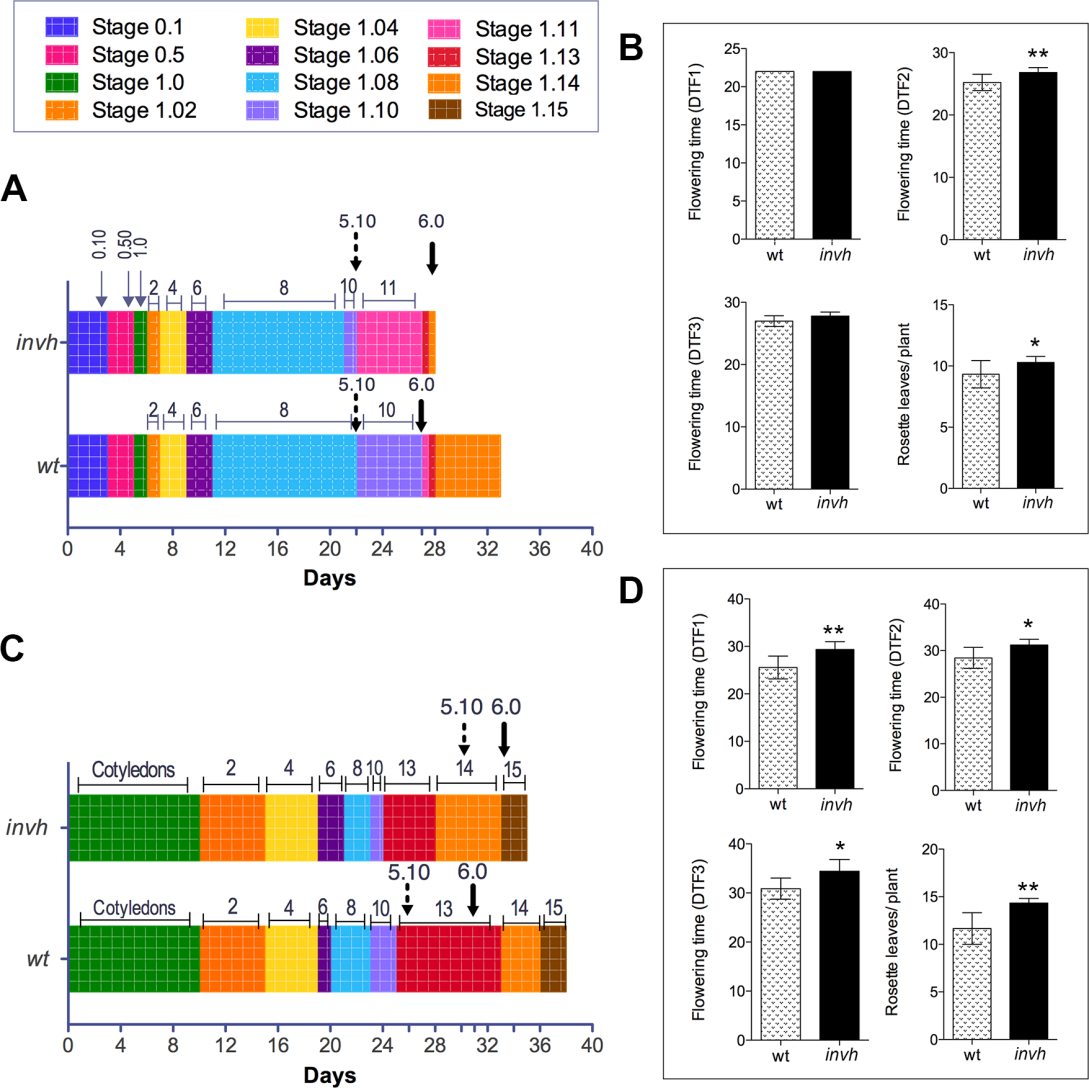
Growth analysis of *invh* Arabidopsis mutant. Comparison of the length of different growth stages of *invh* with respect to wt plants. Growth stage progression was determined in the plate-based early analysis and then transplanting to soil **(A** and **B)** or in the soil-based analysis **(C** and **D)**. **(A)** and **(C)**, analysis according to Boyes et al. (2001) [34]. Arrows define the time (days after sowing) at which *invh* plants reached the following growth stages: 0.10, seed imbibition; 0.50, radicle emergence; 1.0, cotyledons fully opened; 1.02, two rosette leaves > 1 mm in length; 1.04, four rosette leaves > 1 mm in length; 1.06, 1.08, 1.11, 1.13, six, eight, eleven and thirteen rosette leaves (also indicated by numbers on the colors boxes), respectively; 5.10 (dashed arrow), first flower bud perceptible to the eye; 6.0 (black bold arrow), first flower opening. Boxes represent the time elapsed between successive growth stages. Junctions between boxes of different colors indicate the occurrence of a growth stage. Data were collected on a daily basis from three independent experiments (n = 30 plants). **(B** and **D)** Flowering time stages and leaf number corresponding to (**A**) and (**C**) experiment, respectively: DTF1, time when the floral buds became visible in the center of the rosette (equivalent to 5.10 in **A** and **C**); DTF2, time when the main shoot was 1 cm long; DTF3, time when the first flower opened (equivalent to 6.0 in **A** and **C**). The number of rosette leaves per plant was determined when the main shoot was 1 cm long (DTF2). Average of 15 plants ± SD, from two independent experiments. Significant statistically differences (t-test) are indicated with asterisks: * (P ≤ 0.05); ** (P ≤ 0.01).

**Fig 4:**
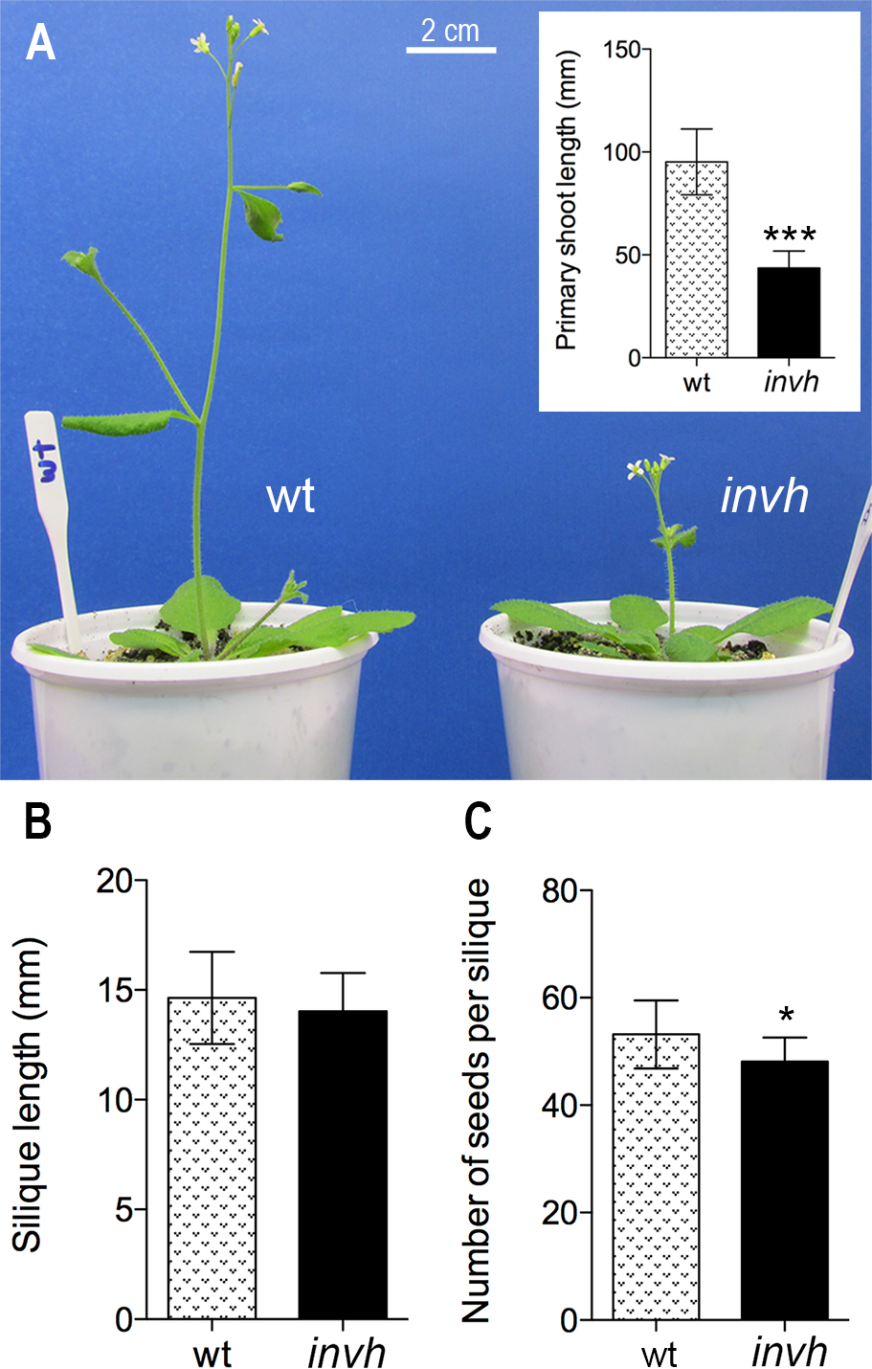
Shoot and silique lengths and number of seeds per silique in Arabidopsis *invh* plants. (**A**) Wild-type and *invh* plants at 29 days after sowing. Inset shows the primary shoot lengths measured 29 days after sowing. (**B**) Mature silique length and (**C**) Number of seeds *per* mature silique. Average of 15 plants ± SD, from two independent experiments. Significant statistically differences (t-test) are indicated with asterisks: * (P < 0.05); *** (P < 0.001).

To investigate A/N-InvH function, we analyzed the phenotype of Arabidopsis mutant plants (*invh*). Homozygous plants for T-DNA insertion in *A/N-InvH* were first identified by PCR amplification, using T-DNA-flanking primers (RP/LP and RP/LB), according to http://signal.salk.edu/tdnaprimers.2.html (S1 Fig). Figs 3 and 4 summarize *invh* phenotype description. Growth stages of *invh* plants were compared to those of wt plants in both plate- and soil- based conditions. The time when the different organs emerged after transferring seeds to the growth chamber was registered according to Boyes et al. (2001) [34] (Fig 3). No delay was observed in the first developmental stages (0.1 to 1.07). The emergence of the floral bud and the first flower opening were the most affected stages. In plate-based growth (Fig 3A and 3B), a significant difference was observed in *invh* plants that reached the first flower bud (stage 5.10/ DTF1) and open the first flower (stage 6.0/DTF3) when they had one and two leaves more, respectively, than wt plants (Fig 3A). In soil-grown plants (Fig 3C and 3D), those differences became more evident since the first bud of wt plants became visible (stage 5.10/DTF1) after 23 days, with 8 leaves, and the first flower opened (stage 6.0/DTF3) after 28 days (11-leaves plants), while *invh* mutant plants reached the stage 5.10 in 29 days, with 13 leaves, and started flowering on day 33 (Fig 3C).

The germination kinetics of *invh* seeds was similar to that of wt seeds (S4 Fig). On the other hand, while root development in *invh* and wt seedlings was similar (S5 Fig), a significantly reduced primary shoot growth was registered in *invh* seedlings, when compared to that of wt (Fig 4A), reaching the maximum difference at day 29 after sowing. The length of siliques was similar in both plants (Fig 4B), though a slightly lower number of seeds per silique was obtained from the *invh* mutant (Fig 4C). Nonetheless, neither aborted ovules nor embryo lethal phenotypes were observed.

### *A/N-InvH* promoter is markedly active in reproductive tissues

**Fig 5:**
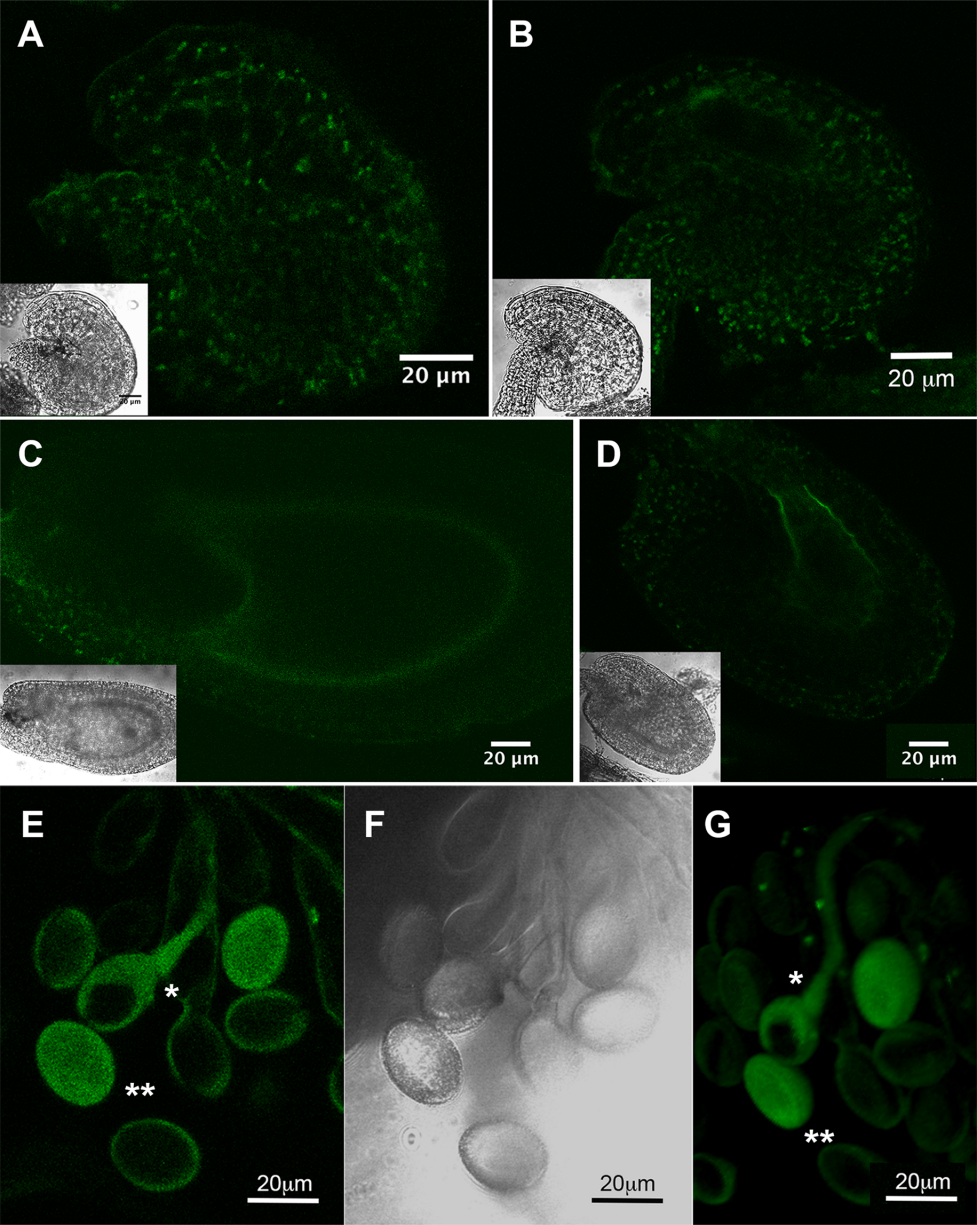
*A/N-InvH* promotor is expressed in *Arabidopsis thaliana* reproductive tissues. The promoter region of *A/N-InvH* (1.9 kbp upstream the translation start codon) fused to *gfp* was introduced into *Arabidopsis* wt by floral dip. **(A-D)** Female gametophytes: **(A)** and **(B)**, unfertilized ovules; **(C)** and **(D)**, fertilized ovules. The segregation between *ProInvH*::*gfp* (green fluorescent pollen grains) and wt (non-fluorescent pollen grains) can be clearly observed. **(E)** Germinating (*) and non-germinating (**) pollen on the stigma. **(F)** Bright-field images corresponding to **(E)**. **(G)** 3D reconstruction of **(E)** confocal images.

To better understand A/N-InvH physiological function, we investigated *A/N-InvH* expression *in vivo*, by generating *ProinvH*::*gfp* transgenic lines and analyzing the *gfp* expression pattern in different tissues by confocal microscopy. GFP fluorescence was markedly high in male and female gametophytes (Fig 5). More specifically, in unfertilized female gametophyte the expression of the reporter gene was found in maternal tissue (Fig 5A) and at the micropylar end of the embryo sac (Fig 5B). In fertilized ovules, *gfp* expression was detected in the endosperm region (Fig 5C and 5D). Remarkably, a strong fluorescent signal was observed in mature and germinated pollen (Fig 5E–5G). Meta-profile analyses of *A/N-InvH* expression using Arabidopsis GENEVESTIGATOR Web-browser are consistent with our results regarding inflorescence components (S6 Fig). We could not observed fluorescence signals in leaves or shoots; however, a low expression is reported in the GENEVESTIGATOR meta-profile analysis (S7 and S8 Figs).

### Mitochondria functionality is compromised in *invh* roots

**Fig 6:**
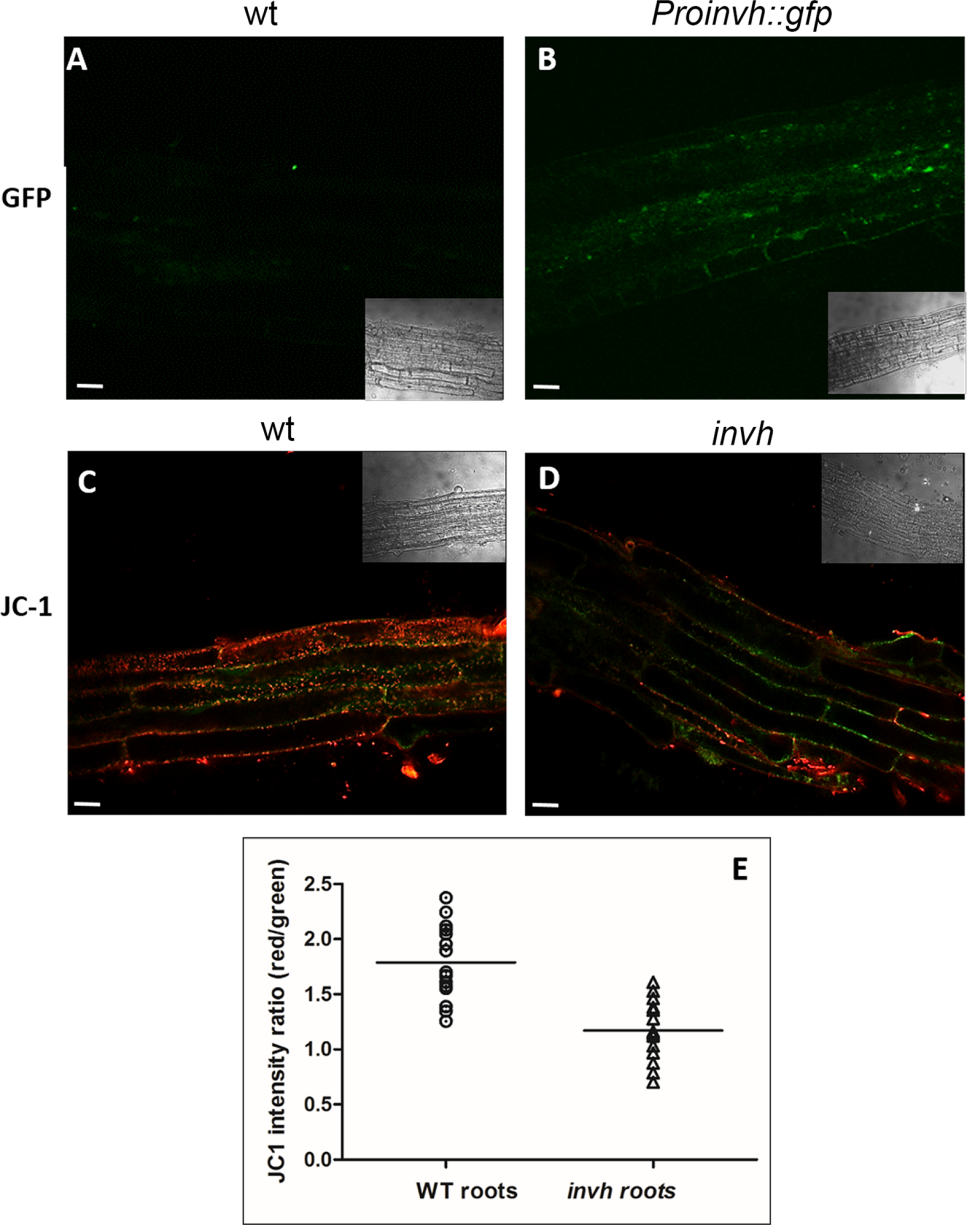
*A/N-InvH* expression in Arabidopsis roots is involved in mitochondrial membrane potential. **(A)** wt plants (control, showing autofluorescence background); **(B)** *gfp* expression in the root elongation zone of transgenic Arabidopsis *ProInvH*::*gfp* plants, analyzed in a confocal microscope; **(C)** wt root stained with JC-1 dye; **(D)** Root from *invh* plants stained with JC-1-dye. **(E)** Dispersion graph showing the values of the red/green ratio of JC-1 fluorescence recorded in wt (circles) and *invh* mutant roots (triangles) by ImageJ software. The horizontal lines indicate the mean values for wt (1.8) and *invh* (1.3) roots. A total of 15 and 16 roots were analyzed from wt and *invh* plants, respectively. Representative images are shown. Scale bar: 20 μm.

**Fig 7:**
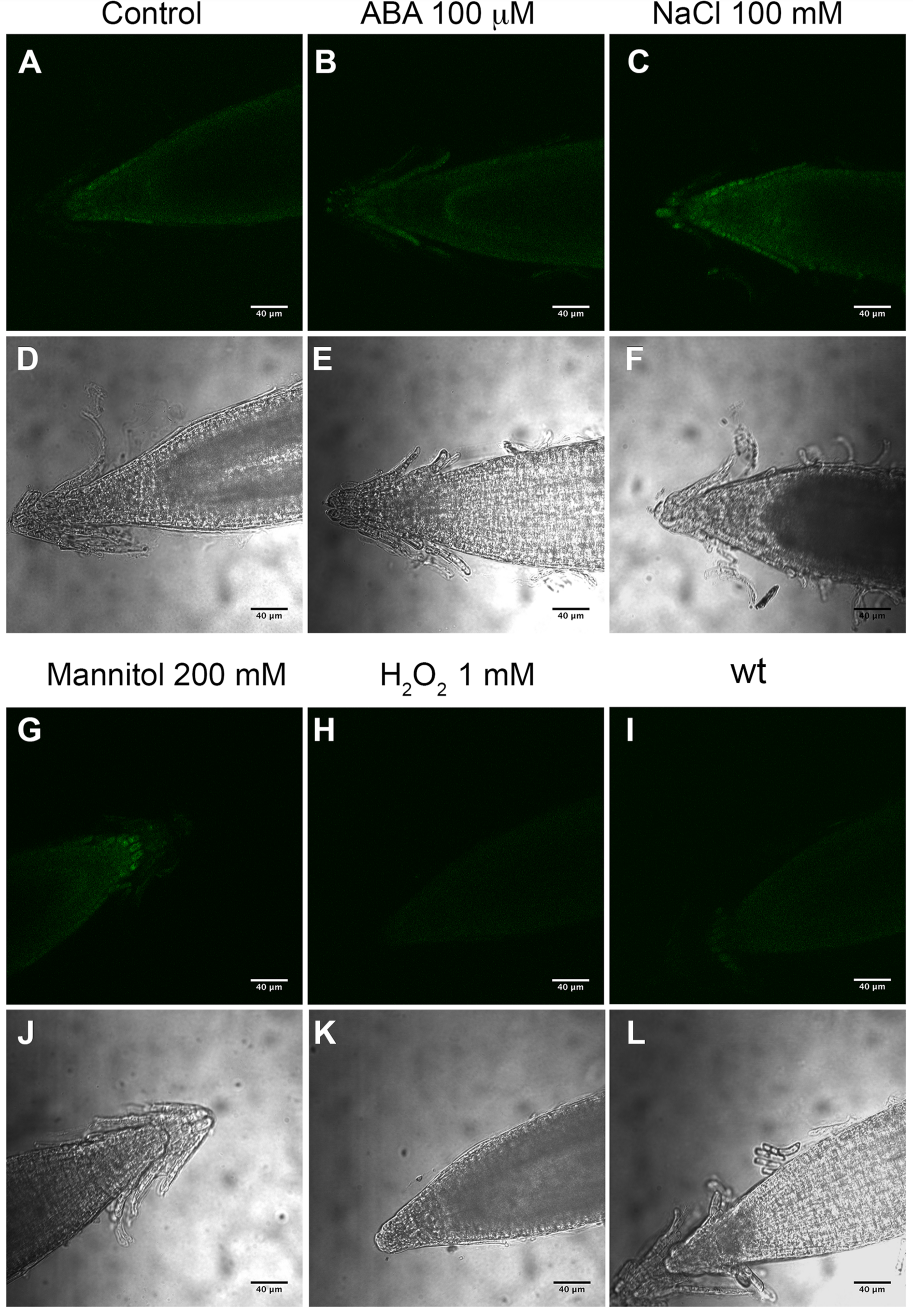
*A/N*-*InvH* expression is induced by salt, osmotic and ABA treatments. Seven-day-old Arabidopsis transgenic seedlings, expressing *ProInvH*::*gfp* and maintained in vertical plates, were exposed to different treatments. Control, roots from untreated seedling, (**A** and **D**); roots from seedlings exposed to 100 μM ABA **(B** and **E)**, 100 mM NaCl **(C** and **F),** or 200 mM mannitol **(G** and **J)** for 24 h, or to 1 mM H_2_O_2_ in MS solution for 30 min **(H** and **K)**. Root from wt seedling **(I** and **L)** was included to visualize tissue autofluorescence. (**A**-**C** and **G**-**I**), are images collected using the confocal parameters setting as for control conditions. **(D-F** and **J-L)**, are bright-field images.

In transgenic *ProinvH*::*gfp* plants, *gfp* expression was clearly detected, in both the elongation (Fig 6B) and the meristematic (Fig 7A) root zones. To learn whether A/N-InvH could be contributing to the mitochondrial functional status, roots of wt and *invh* mutant plants were stained with the membrane potential indicator JC-1 dye [47,48] and examined for red and green fluorescence (Fig 6C and 6D). Roots from wt plants (Fig 6C) exhibited cells with high mitochondrial potential, and JC-1 formed intense red fluorescent complexes. Conversely, a lower mitochondrial potential was evidenced in roots of *invh* plants using JC-1 (Fig 6D), which remained in its monomeric form, displaying green fluorescence. JC-1 intensity red/green ratio is shown in Fig 6E.

To investigate the effect of abiotic stress on *A/N-InvH* expression in roots, transgenic plants carrying the *ProinvH*::*gfp* fusion were exposed to NaCl, mannitol, H_2_O_2_ or ABA (considered a plant stress hormone) [52]. As shown in Fig 7, *A/N-InvH* is mainly transcribed in the lateral and columella root cap from untreated plants, and transcription increases twofold after exposure to 100 mM NaCl for 24 h (Fig 7C, S9 Fig). The mannitol treatment also resulted in a nearly 100% expression increment in the columella root cap (Fig 7B, S9 Fig). No effect was detected with the hydrogen peroxide treatment (Fig 7G, S9 Fig). Notably, the presence of ABA did not increase total fluorescence (Fig 7B, S9 Fig), but the expression was higher and concentrated in a differentiated cell cap (probably corresponding to the endodermis and/or pericycle).

### The absence of *A/N-invH* prevents ROS formation in roots

**Fig 8:**
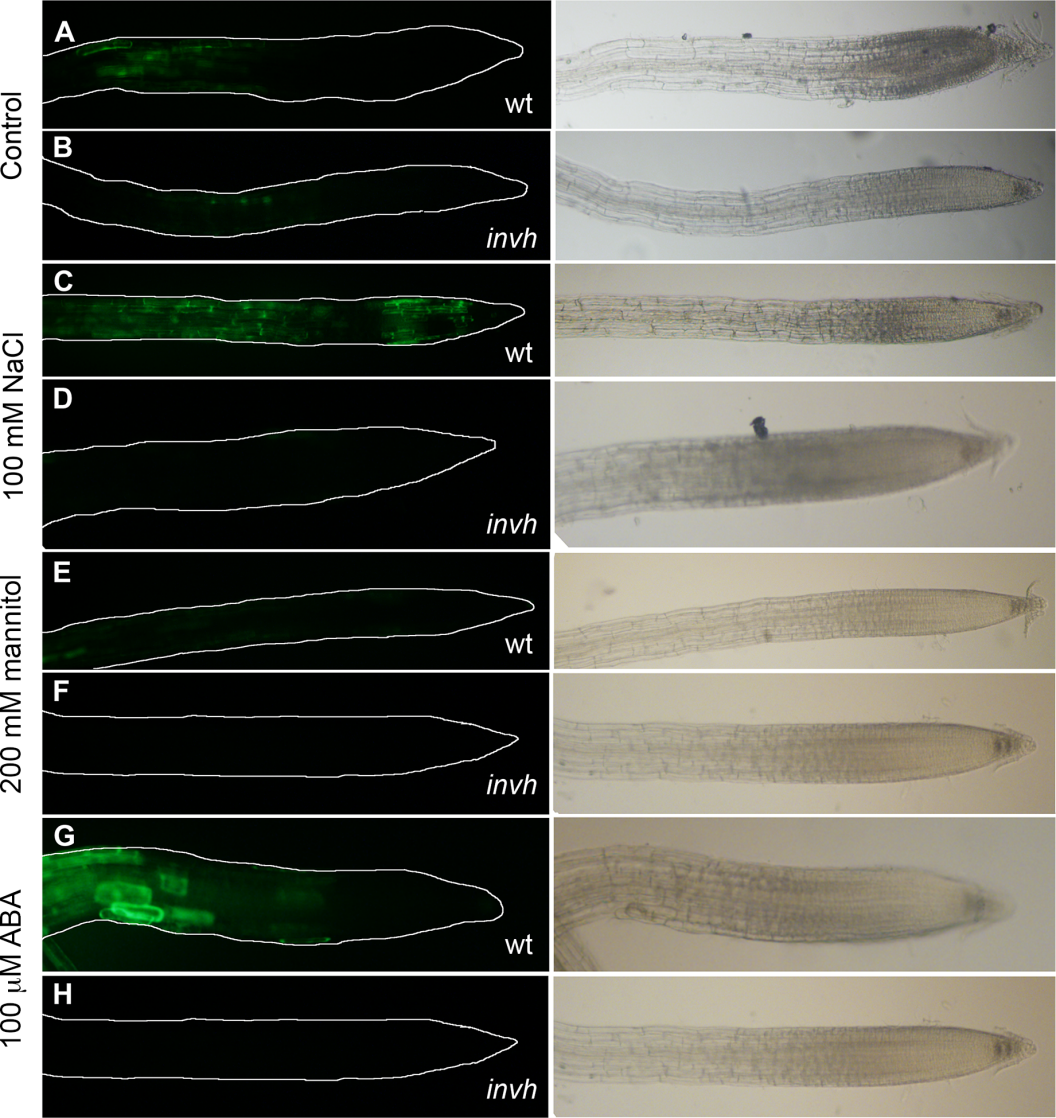
Detection of ROS in Arabidopsis roots from wt and *invh* mutant seedlings subjected to different treatments. Fluorescence in dark-field (left side) and bright-field imaging (right side) show ROS presence and root anatomy, respectively. In dark field, a line was added to delimit the root. Arabidopsis wt and *invh* mutant plants were subjected to different treatments for 30 min. **(A)** and **(B),** control; **(C)** and **(D),** 100 mM NaCl; **(E)** and **(F),** 200 mM mannitol; **(G)** and **(H)** 100 μM ABA. Root images were collected after 10 min incubation with H_2_DCFDA. Fluorescence indicates the presence of ROS.

The compromised mitochondrial functionality in plants lacking A/N-Inv and the recognized role of ROS as key players in the complex signaling network of plants' stress responses led us to investigate the ROS level in *invh* plants. ROS detection was performed with the fluorescent probe H_2_DCFDA [53]. The dye was loaded for 10 min into Arabidopsis wt and *invh* plants, previously treated with 100 mM NaCl, or 200 mM mannitol or 100 μM ABA for 30 min. ROS presence was analyzed under microscope. The small amount of ROS visualized in the elongation zone of wt (control) roots notably increased with the salt treatment and ABA addition (Fig 8A, 8C and 8G). The lack of *A/N-InvH*) prevented ROS formation in all cases (Fig 8B, 8D and 8H).

## Discussion

In the last decade, novel findings on sucrose catabolism have brought to light an unsuspected intricate relationship between organellar sucrose degradation, plant growth and development, stress responses, and the possible interconnection of different signaling pathways. The demonstration of subcellular isoforms of A/N-Inv (in chloroplasts, mitochondria or nuclei) [10] and sucrose synthase (in mitochondria) [11] raised the question about their physiological relevance in organellar function, cell homeostasis, the whole plant physiology and adaptive responses to the prevailing environment.

Although putative mitochondrial A/N-Inv isoforms were predicted from carrot, *Lolium temulentum*, and *A*. *thaliana* sequences [18], the first mitochondrion-targeted A/N-Invs were shown in rice [19] and Jerusalem artichoke tubers [4]. An A/N-Inv (PtrA/NINV), dually targeted to both chloroplasts and mitochondria, has recently been reported in *Poncirus trifoliata* [53]. Phylogenetic analysis based on protein sequences revealed that plant A/N-Invs group into two major clades (called α and β Mitochondrial A/N-Invs’ sequences (including the three Arabidopsis isoforms) ([29,30] and this paper) and chloroplast-targeted A/N-Invs cluster in clade α[18,19,36,22,23,20]. The ubiquity and the multiple isoforms present in each species support the idea that mitochondrial A/N-Invs are common in the plant kingdom, even in distantly related plants, such as the moss *Physcomitrella patens* [54].

In Arabidopsis, two mitochondrial invertases were identified (A/N-InvA and A/N-InvC) [29,30] and their physiological functions studied using knockout plants (*inva* and *invc*, respectively). The present study describes the occurrence of a third mitochondrial isoform (A/N-InvH) (Figs 1 and 2) and the investigation of its localization and function, thus completing the Arabidopsis repertoire of these sucrose hydrolytic proteins located in the matrix of the organelle. The combination of tissue/organ expression and phenotype analysis of the three null mutants revealed unexpected and complex roles for these proteins.

As opposed to A/N-InvA and A/N-InvC, which make a significant contribution to the total A/N-Inv activity [29,30] and whose encoding genes are highly to fairly expressed in every stage of Arabidopsis plant development (S2 Fig), *A/N-InvH* expression could not be detected in most tissues. Notably, *A/N-InvH* is expressed mainly in non-fertilized (maternal tissue, micropylar end of the embryo sac) and fertilized ovules (endosperm region), and male gametophytes (in mature and germinated pollen) (Fig 5). Consistent with those results are the semiquantitative RT-PCR experiments conducted by Vargas et al. (2008) [28]. We also show that *A/N-InvH* is transcribed in roots in both the elongation and the apical meristem zones (Figs 6 and 7A).

A general feature of *inva* [29], *invc* [30] and *invh* (Fig 4) knockout mutant plants is a severe reduction in the shoot growth, as compared to wt plants. On the one hand, *inva* and *invc* share an impaired growth phenotype [29,30] and, on the other, both *invc* and *invh* plants present a significant delay in flowering stages (emergence of the first flower bud and first flower opening) [30] (Figs 3 and 4). Moreover, the differential roles of A/N-InvA, -InvC and -InvH are supported by particular characteristics, evidenced in the respective knockout mutants. Only *inva* showed smaller root length (44% shorter than the wt roots), which was ascribed to typical phenotype for plants suffering from oxidative stress [29], and only *invc* showed delayed germination (S4 Fig) [30] related to hormone (ABA/gibberellic acid) balance modulation by A/N-InvC. This isoform was proposed to be involved in ABA signaling and/or activating GA signal transduction pathways [30]. Moreover, despite A/N-InvH being mainly expressed in reproductive tissues (Fig 5), *invh* plants showed neither aborted ovules nor an embryo lethal phenotype; however, a low number of seeds per silique was determined (Fig 4), a trait that was not shared by *invc* (mutant that also presented flowering delay) [30]. Taken together, these results led us to conclude that the three isoforms that can hydrolyze sucrose in the mitochondrial matrix are not redundant. These findings together constitute an important contribution to understanding the complex communication network between mitochondria, cytosol (the place where sucrose is synthesized), and other cell compartments to regulate plant growth and development.

The mitochondrial functional status of *invh* plants was investigated in roots. The reduction registered in mitochondrial potential (Fig 6) suggests lower respiration and dysfunctional mitochondria [47]. This was also demonstrated for *invc* and *inva* plants (50% reduction in oxygen consumption [30]). In other words, the lack of any of the three mitochondrial isoforms directly translates into prominent respiration impairment, indicating that they function independently. It is likely that A/N-Inv activity mainly controls respiration rate and ATP generation [4]. Invertases could provide glucose to hexokinase, associated with the outer mitochondrial membrane, producing ADP, which, in turn, could be used for ATP regeneration, supporting oxidative phosphorylation [55,29]. The existence of plant heterogeneous mitochondrial populations has been described with respect to morphology and behavior in different plant tissues and in cells at different developmental stages [56,57]. This all seems to indicate that each mitochondrial A/N-Inv, in addition to contributing to the respiration process, may be fulfilling different functions, probably, in specific mitochondrial populations.

ROS play an essential role in regulating numerous responses to biotic and abiotic stresses in plants. The fine balance between the production and scavenging of ROS is disrupted after environmental stresses that induce oxidative damage [58,59]. A comprehensive study showed that different stressors (including salt and osmotic treatments and ABA addition) modulate the transcription of a range of genes encoding mitochondrial proteins. Besides, the interconnection of mitochondrial ROS and ABA signaling has not, as yet, been elucidated [60,61]. *A/N-InvH* transcription in the Arabidopsis root apical meristem zone was increased twofold in plants subjected to salt or osmotic stress (Fig 7, S9 Fig). Nonetheless, no effect was detected in the presence of H_2_O_2_ (S9 Fig). A particular response was observed in the presence of ABA that raised *A/N-InvH* expression in a differentiated cell cap but not in the whole root. The visualization of ROS production in wt plant roots was evident after salt or ABA treatment. Surprisingly, the absence of A/N-InvH prevented ROS formation in Arabidopsis roots of control and treated plants (Fig 8). We hypothesize that, even when plants are not stressed, A/N-InvH may be involved in ROS production, related to both root development and stress defense. The importance of ROS in root growth has been well described, particularly in the elongation zone and tip [62]. The association of ROS with mitochondrial function disruption and their participation in biological processes has been widely investigated [58, 63, 64–66]. A different behavior to that of *invh* was reported in *inva* plants, where ROS levels increased after oxidative stress, and A/N-InvA was proposed to be part of the antioxidant system involved in cellular ROS homeostasis [29]. Differential responses in ROS production were reported in Arabidopsis mitochondrial mutants displaying different ABA sensitivity, as ROS content increased in some mutants and decreased in others [63, 66–68]. In addition, it was shown that sugars indirectly contribute to antioxidative mechanisms and are involved in direct ROS quenching in different organelles [69]. An interaction between ROS and sugar signaling pathways is expected, pointing towards sucrose and glucose functioning as central integrating regulatory molecules that control endogenous developmental and metabolic cues [5]. In this context, the mitochondrial A/N-Inv system can be a pivotal actor in operating a complex regulatory network that includes ROS signaling, as well as plant-specific hormone and stress-related signaling pathways [70].

The occurrence of three A/N-Invs localized in mitochondria has presented an intricate but fascinating puzzle. The challenge is to provide experimental evidence to address, among other questions, how the mitochondrial invertase system integrates into intermediary metabolism, which is the physiological function of these isoforms inside the organelle and in the communication with other subcelullar compartments, how plant growth and development and ROS production are coordinated and how so many signals are interconnected.

## Conclusions

This study provides the last piece to complete the A/N-Inv mitochondrial repertoire in a plant species. We demonstrated that A/N-InvH is involved in growth (e.g., shoot elongation) and participates in developmental processes (e.g., reproductive tissues’ development), playing roles other than those of A/N-InvA and–InvC. Undoubtedly, the three isoforms are likely to be relevant in sustaining functional mitochondria and regulating the organellar sucrose/hexoses levels. Those sugars and ROS participate in the intricate signaling network that links the key functions of the different compartments of plant cells. It is time to start deciphering the puzzle.

## Supporting information

S1 Table *Arabidopsis thaliana A/N-Inv* genes coding for mitochondrion-target proteins. (PDF)

S2 Table Bioinformatic analysis to determine the subcellular localization of Arabidopsis A/N-InvH. MitoProt II (v1.101), Target P1.1, Protein Prowler, and PSORT softwares were used to predict the subcellular localization of the A/N-InvH protein. M, mitochondria; C, chloroplasts.
(PDF)

S1 Fig Analysis of Arabidopsis homozygous mutant *invh* genotype. Lines provided by TAIR (SALK_103674.18.70.x, SALK T-DNA homozygous knockout line for At3g05820) were analyzed using the primers according to SALK T_DNA primer design (LP, TTGGTGGCGTCCATAGAGTAC; RP, TGGTTTCGAGGGTGTTAAGTG; and LB, ATTTTGCCGATTTCGGAAC).
**(a) Schematic representation of the T-DNA insertion site in the mutant used in this study and *A/N-InvH* gene (locus At3g05820) structure.** Exons **(**black bars) and introns (black lines). T-DNA insertion site is depicted as a white box in the second exon (SALK_103674.18.70.x, knockout mutation line, called *invh*) and the primer positions (RP, LP and LB, http://signal.salk.edu/tdnaprimers.2.html) are indicated with arrows. **(b)** Genotypic characterization of *invh* by PCR. Homozygosis of the mutant line *invh* used in this study was confirmed by PCR analysis using genomic DNA from Arabidopsis Col-0 (wild-type, wt) and *invh* mutant, and the primer pairs RP/LP and RP/LB. Amplification products were separated by electrophoresis on 1% agarose gels and visualized after ethidium bromide staining.
(PDF)

S2 Fig Comparison of Arabidopsis *A/N-InvA* (At1g56560), *A/N-InvC* (locus At3g06500) and *A/N-InvH* (At3g05820) gene expression at different stages of plant development. Expression values and standard deviations were calculated from all microarrays annotated for each particular stage. Analysis was performed from the Arabidopsis GENEVESTIGATOR browser (www.genevestigator.com) [51]
(PDF)

S3 Fig Subcellular localization of the protein product of the A/N-InvH gene. (a) A 795-bp *A/N-InvH* fragment encoding 265 amino acids from the N-terminal was in-frame fused upstream the *gfp* reporter gene driven by CaMV 35S promoter. Position of the putative organelle transit peptide in the N-terminal (1–43) is indicated. (b-g) GFP expression in *Nicotiana benthamiana* leaves. The *35S*::*A/N-invh*::*gfp* construct was cloned in pCambia1302 and used to transiently transform *N*. *benthamiana* leaves via *Agrobacterium tumefaciens* GV3101 [Llave et al., Proc Natl Acad Sci USA 97:13401–13406 (2000)]. (b) and (c) images obtained from an epifluorescence microscope. Black arrows indicate chlorophyll autofluorescence. (d) and (f) GFP fluorescence analyzed by confocal microscopy is located in mitochondria and absent in chloroplasts. White arrows indicate a stomata. (e) and (g) Bright field.
(PDF)

S4 Fig Germination curve of Arabidopsis *invh* mutant and wt seeds. Seeds were surface-sterilized and plated on MS-1X containing 0.05% Mes-KOH (pH 5.7), 1% sucrose as carbon source and 0.8% agar. To break the dormancy, plates were maintained at 4°C for 3 days in the dark prior to germination. Then seeds were incubated under controlled conditions of photoperiod and temperature (16 h/8 h, light/dark, 22±1°C) and germination was registered under stereoscopic microscope to evaluate the visible radicle tip [34]. Three independent plates with one hundred wt or *invh* seeds were analyzed with. Average of 300 seeds ± SD.
(PDF)

S5 Fig Root length of *invh* mutant and wt Arabidopsis seedlings. Root length was evaluated on 7 days-old seedlings growing on vertical plates on MS medium (pH 5.7), solidified with 0.8% agar. To scale and quantify the root length, plates were photographed with a ruler on a side. Images were analyzed using ImageJ software. Statistical analysis was performed using Prism software. Data were collected from four biological replicates. Vertical bars represent the SE for n = 24.
(PDF)

S6 Fig Meta-profile analysis of *Arabidopsis thaliana* mitochondrial *A/N-Inv* gene expression. *A/N-InvA* (At1g56560, *A/N-InvC* (At3g06500), and *A/N-InvH* (At3g05820) expression analysis was performed from the GENEVESTIGATOR browser (www.genevestigator.com). Inflorescence category data were expanded.
(PDF)

S7 Fig Meta-profile analysis of *Arabidopsis thaliana* mitochondrial *A/N-Inv* gene expression. *A/N-InvA* (At1g56560, *A/N-InvC* (At3g06500), and *A/N-InvH* (At3g05820) expression analysis was performed from the GENEVESTIGATOR browser (www.genevestigator.com). Shoot category data were expanded.
(PDF)

S8 Fig Meta-profile analysis of *Arabidopsis thaliana* mitochondrial *A/N-Inv* gene expression. *A/N-InvA* (At1g56560, *A/N-InvC* (At3g06500), and *A/N-InvH* (At3g05820) expression analysis was performed from the GENEVESTIGATOR browser (www.genevestigator.com). Root category data were expanded.
(PDF)

S9 Fig Quantification of GFP fluorescence in root caps corresponding to Fig 7 images. GFP signals were quantified by confocal microscopy using ImageJ and correspond to images of *A/N*-*InvH* gene expression after salt (NaCl 100 mM), mannitol (200 mM), ABA (100 μM), and oxygen peroxide (1 mM) treatments. Seven days-old transgenic seedlings of wt plants expressing *A/N-InvH* promoter fused to GFP were exposed to 100 μM ABA, 100 mM NaCl or 200 mM mannitol for 24 h, on vertical plates with MS medium. Treatment with 1 mM H_2_O_2_ was performed for 30 min in MS solution. After treatments, GFP fluorescence was observed in a confocal microscope. All images were collected using the confocal parameters setting for control conditions. Control corresponds to non-transgenic wt plants to visualize autofluorescence. In all images equal areas were measured as described by in Martin et al. (2014) [46].
(PDF)

S10 Fig Mitochondrial *A/N-Inv* expression in different segments of the primary stem of Arabidopsis plants. Expression data correspond to Arabidopsis primary stem (DataSet Record: GDS2895) from full-genome microarrays [Ehlting et al., Plant J 42:618–640 (2005)] deposited in GEO public data repository [Edgar et al., Nucleic Acids Res 30:207–210 (2002)]. (A-C) Expression of the three mitochondrial *A/N-Inv* genes [*A/N-InvH* (At3g05820), *A/N-InvA* (At1g56560) and *A/N-InvC* (At3g06500)] in two stages of development (5 cm, light green; 10 cm, green). Stems were cut in sections (from the apical meristem) indicated in the pink boxes.
(PDF)

## Abbreviations

ABA: abscisic acid
A/N-Inv: Alkaline/Neutral-Invertase
At-A/N-InvH: *Arabidopsis thaliana* Alkaline/Neutral-Invertase H
*invh*: Arabidopsis mutant lacking alkaline/neutral-invertase H
H_2_DCFDA: 2',7'-dichlorodihydrofluorescein diacetate
MS: Murashige-Skoog
ROS: Reactive Oxygen Species
wt: Arabidopsis wild-type plants, ecotype Columbia 0 (Col-0)
