# Supplementary material for: The riddle of mitochondrial alkaline/neutral invertases: A novel Arabidopsis isoform mainly present in reproductive tissues and involved in root ROS production
Source: PLoS One. 2017 Sep 25;12(9):e0185286. doi: 10.1371/journal.pone.0185286 (PMC5612693; doi:10.1371/journal.pone.0185286)
Supplement: S1 Table — (PDF) [file pone.0185286.s001.pdf]

Supporting information

The riddle of mitochondrial alkaline/neutral invertases: A novel Arabidopsis isoform mainly present in reproductive tissues and involved in root ROS production.

Marina E. Battaglia, María Victoria Martin, Leandra Lechner, Giselle M.A. Martínez-Noël, Graciela L. Salerno

S1 Table. *Arabidopsis thaliana A/N-Inv* genes coding for mitochondrion-target proteins.

**Name** (Reference)	**Locus ID**	**Location**	**Exon/Intron number**	**Transcript length** (bp)	**Protein length** (amino acid residues)	**UNIPROT**
A/N-InvH (this study)	At3g05820.2	**Chr 3** 1,732,988-1,735,765 reverse strand	6/5	2,196	633	Q84JL5
A/N-InvA [28]	At1g56560.1	**Chr 1** 21,192,137-21,195,166 forward strand	6/6	2,525	616	Q9FXA8
A/N-InvC [29]	At3g06500.1	**Chr 3** 2,011,993-2,015,831 forward strand	6/5	2,671	664	B9DFA8
