# Supplementary material for: The riddle of mitochondrial alkaline/neutral invertases: A novel Arabidopsis isoform mainly present in reproductive tissues and involved in root ROS production
Source: PLoS One. 2017 Sep 25;12(9):e0185286. doi: 10.1371/journal.pone.0185286 (PMC5612693; doi:10.1371/journal.pone.0185286)
Supplement: S2 Table — MitoProt II (v1.101), Target P1.1, Protein Prowler, and PSORT softwares were used to predict the subcellular localization of the A/N-InvH protein. M, mitochondria; C, chloroplasts. (PDF) [file pone.0185286.s002.pdf]

Supporting information

The riddle of mitochondrial alkaline/neutral invertases: A novel Arabidopsis isoform mainly present in reproductive tissues and involved in root ROS production.

Marina E. Battaglia, María Victoria Martin, Leandra Lechner, Giselle M.A. Martínez-Noël, Graciela L. Salerno

S2 Table. Bioinformatic analysis to determine the subcellular localization of Arabidopsis A/N-InvH. MitoProt II (v1.101), Target P1.1, Protein Prowler, and PSORT softwares were used to predict the subcellular localization of the A/N-InvH protein. M, mitochondria; C, chloroplasts.

**Software**	**Targeted organelle**	**Cleavage site** (amino acid residue)	**Cleaved sequence**	**Probability of export to organelle**
MitoProt II (v1.101)	M	44	MNAITFLGNSTMIPSQCILRAF TRISPSKYIRDTSFRSYPSRF	0.972
TargetP 1.1	C	-	-	0.847
Protein Prowler	C	-	-	0.900
PSORT (v6.4)	M	53	YRNADS	0.499
