# Supplementary material for: The riddle of mitochondrial alkaline/neutral invertases: A novel Arabidopsis isoform mainly present in reproductive tissues and involved in root ROS production
Source: PLoS One. 2017 Sep 25;12(9):e0185286. doi: 10.1371/journal.pone.0185286 (PMC5612693; doi:10.1371/journal.pone.0185286)
Supplement: S1 Fig — Lines provided by TAIR (SALK_103674.18.70.x, SALK T-DNA homozygous knockout line for At3g05820) were analyzed using the primers according to SALK T_DNA primer design (LP, TTGGTGGCGTCCATAGAGTAC; RP, TGGTTTCGAGGGTGTTAAGTG; and LB, ATTTTGCCGATTTCGGAAC). (a) Schematic representation of the T-DNA insertion site in the mutant used in this study and A/N-InvH gene (locus At3g05820) structure. Exons (black bars) and introns (black lines). T-DNA insertion site is depicted as a white box in the second exon (SALK_103674.18.70.x, knockout mutation line, called invh) and the primer positions (RP, LP and LB, http://signal.salk.edu/tdnaprimers.2.html) are indicated with arrows. (b) Genotypic characterization of invh by PCR. Homozygosis of the mutant line invh used in this study was confirmed by PCR analysis using genomic DNA from Arabidopsis Col-0 (wild-type, wt) and invh mutant, and the primer pairs RP/LP and RP/LB. Amplification products were separated by electrophoresis on 1% agarose gels and visualized after ethidium bromide staining. (PDF) [file pone.0185286.s003.pdf]

Supporting information

The riddle of mitochondrial alkaline/neutral invertases: A novel Arabidopsis isoform mainly present in reproductive tissues and involved in root ROS production.

Marina E. Battaglia, María Victoria Martin, Leandra Lechner, Giselle M.A. Martínez-Noël, Graciela L. Salerno

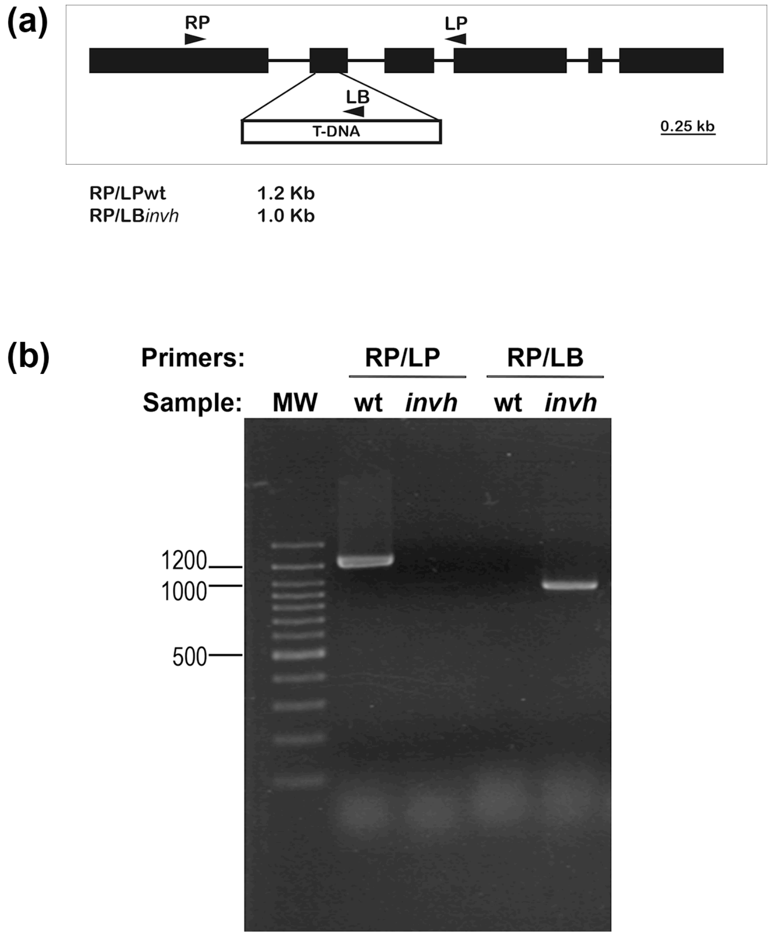

S1 Fig. Analysis of Arabidopsis homozygous mutant *invh* genotype. Lines provided by TAIR (SALK_103674.18.70.x, SALK T-DNA homozygous knockout line for At3g05820) were analyzed using the primers according to SALK T_DNA primer design (LP, TTGGTGGCGTCCATAGAGTAC; RP, TGGTTTCGAGGGTGTTAAGTG; and LB, ATTTTGCCGATTTCGGAAC).

(a) Schematic representation of the T-DNA insertion site in the mutant used in this study and *A/N-InvH* gene (locus At3g05820) structure. Exons (black bars) and introns (black lines). T-DNA insertion site is depicted as a white box in the second exon (SALK_103674.18.70.x, knockout mutation line, called *invh*) and the primer positions (RP, LP and LB,

http://signal.salk.edu/tdnaprimers.2.html) are indicated with arrows. **(b)** Genotypic characterization of *invh* by PCR. Homozygosis of the mutant line *invh* used in this study was confirmed by PCR analysis using genomic DNA from Arabidopsis Col-0 (wild-type, wt) and *invh* mutant, and the primer pairs RP/LP and RP/LB. Amplification products were separated by electrophoresis on 1% agarose gels and visualized after ethidium bromide staining.
