# Supplementary material for: The riddle of mitochondrial alkaline/neutral invertases: A novel Arabidopsis isoform mainly present in reproductive tissues and involved in root ROS production
Source: PLoS One. 2017 Sep 25;12(9):e0185286. doi: 10.1371/journal.pone.0185286 (PMC5612693; doi:10.1371/journal.pone.0185286)
Supplement: S2 Fig — Expression values and standard deviations were calculated from all microarrays annotated for each particular stage. Analysis was performed from the Arabidopsis GENEVESTIGATOR browser (www.genevestigator.com) [51] (PDF) [file pone.0185286.s004.pdf]

Supporting information

The riddle of mitochondrial alkaline/neutral invertases: A novel Arabidopsis isoform mainly present in reproductive tissues and involved in root ROS production.

Marina E. Battaglia, María Victoria Martin, Leandra Lechner, Giselle M.A. Martínez-Noël, Graciela L. Salerno

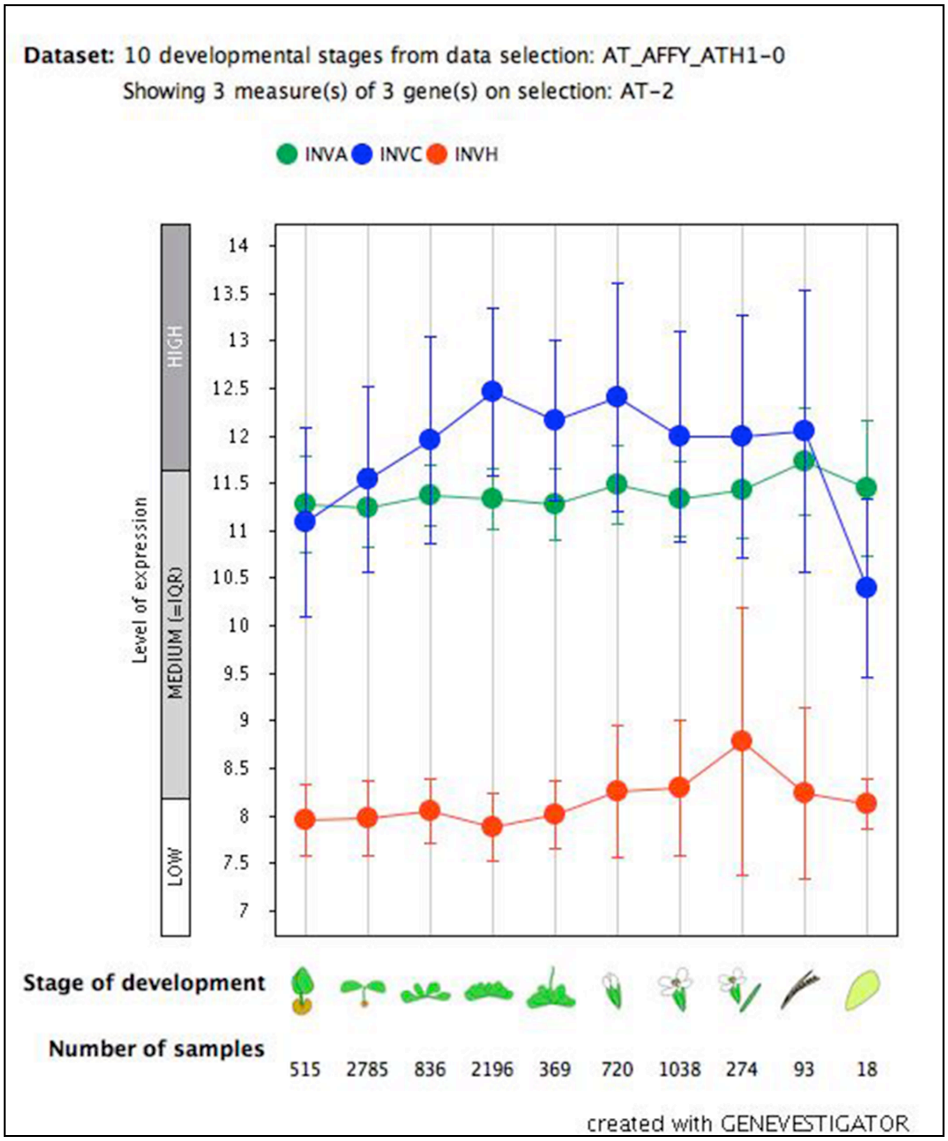

S2 Fig. Comparison of Arabidopsis *A/N-InvA* (At1g56560), *A/N-InvC* (locus At3g06500) and *A/N-InvH* (At3g05820) gene expression at different stages of plant development. Expression values and standard deviations were calculated from all microarrays annotated for each particular stage. Analysis was performed from the Arabidopsis GENEVESTIGATOR browser (www.genevestigator.com) [51].
