# Supplementary material for: The riddle of mitochondrial alkaline/neutral invertases: A novel Arabidopsis isoform mainly present in reproductive tissues and involved in root ROS production
Source: PLoS One. 2017 Sep 25;12(9):e0185286. doi: 10.1371/journal.pone.0185286 (PMC5612693; doi:10.1371/journal.pone.0185286)
Supplement: S3 Fig — (a) A 795-bp A/N-InvH fragment encoding 265 amino acids from the N-terminal was in-frame fused upstream the gfp reporter gene driven by CaMV 35S promoter. Position of the putative organelle transit peptide in the N-terminal (1–43) is indicated. (b-g) GFP expression in Nicotiana benthamiana leaves. The 35S::A/N-invh::gfp construct was cloned in pCambia1302 and used to transiently transform N. benthamiana leaves via Agrobacterium tumefaciens GV3101 [Llave et al., Proc Natl Acad Sci USA 97:13401–13406 (2000)]. (b) and (c) images obtained from an epifluorescence microscope. Black arrows indicate chlorophyll autofluorescence. (d) and (f) GFP fluorescence analyzed by confocal microscopy is located in mitochondria and absent in chloroplasts. White arrows indicate a stomata. (e) and (g) Bright field. (PDF) [file pone.0185286.s005.pdf]

Supporting information

The riddle of mitochondrial alkaline/neutral invertases: A novel Arabidopsis isoform mainly present in reproductive tissues and involved in root ROS production.

Marina E. Battaglia, María Victoria Martin, Leandra Lechner, Giselle M.A. Martínez-Noël, Graciela L. Salerno

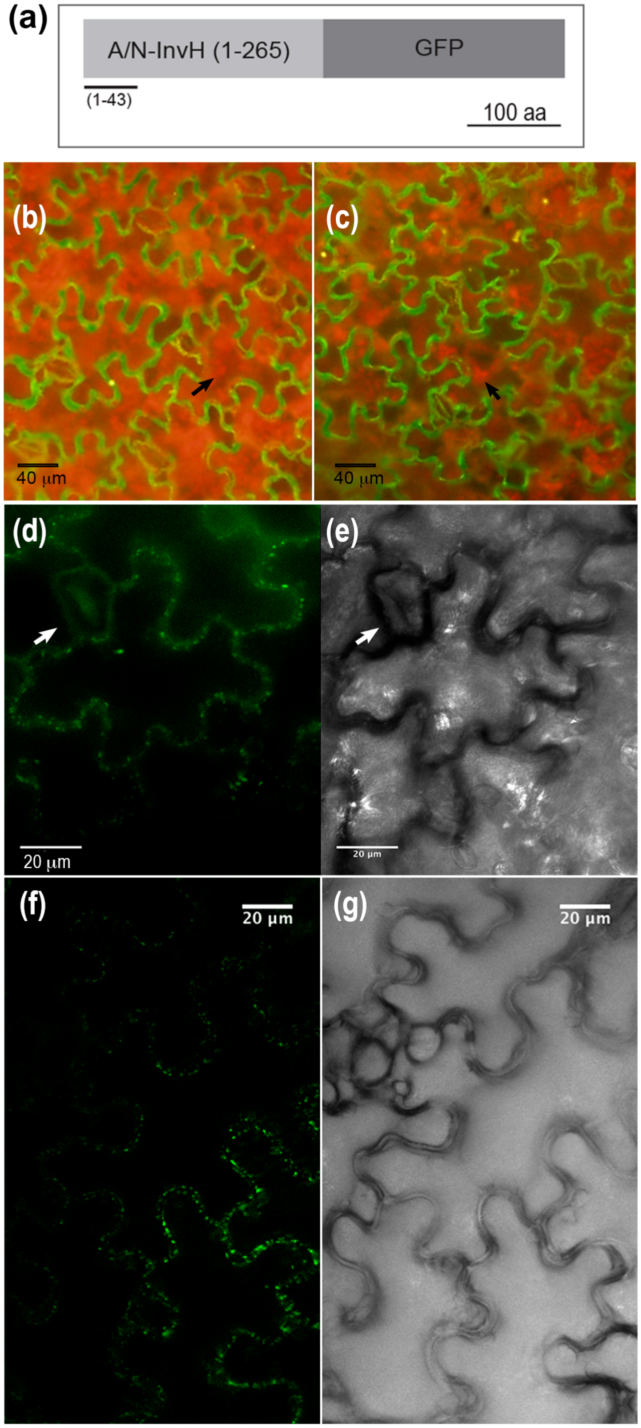

S3 Fig. Subcellular localization of the protein product of the A/N-InvH gene.

(a) A 795-bp *A/N-InvH* fragment encoding 265 amino acids from the N-terminal was in-frame fused upstream the *gfp* reporter gene driven by CaMV 35S promoter. Position of the putative organelle transit peptide in the N-terminal (1-43) is indicated. (b-g) GFP expression in *Nicotiana benthamiana* leaves. The *35S::A/N-invh::gfp* construct was cloned in pCambia1302 and used to transiently transform *N. benthamiana* leaves via *Agrobacterium tumefaciens* GV3101 [Llave et al., Proc Natl Acad Sci USA 97:13401-13406 (2000)]. (b) and (c) images obtained from an epifluorescence microscope. Black arrows indicate chlorophyll autofluorescence. (d) and (f) GFP fluorescence analyzed by confocal microscopy is located in mitochondria and absent in chloroplasts.. White arrows indicate a stomata. (e) and (g) Bright field.
