# Supplementary material for: The riddle of mitochondrial alkaline/neutral invertases: A novel Arabidopsis isoform mainly present in reproductive tissues and involved in root ROS production
Source: PLoS One. 2017 Sep 25;12(9):e0185286. doi: 10.1371/journal.pone.0185286 (PMC5612693; doi:10.1371/journal.pone.0185286)
Supplement: S4 Fig — Seeds were surface-sterilized and plated on MS-1X containing 0.05% Mes-KOH (pH 5.7), 1% sucrose as carbon source and 0.8% agar. To break the dormancy, plates were maintained at 4°C for 3 days in the dark prior to germination. Then seeds were incubated under controlled conditions of photoperiod and temperature (16 h/8 h, light/dark, 22±1°C) and germination was registered under stereoscopic microscope to evaluate the visible radicle tip [34]. Three independent plates with one hundred wt or invh seeds were analyzed with. Average of 300 seeds ± SD. (PDF) [file pone.0185286.s006.pdf]

Supporting information

The riddle of mitochondrial alkaline/neutral invertases: A novel Arabidopsis isoform mainly present in reproductive tissues and involved in root ROS production.

Marina E. Battaglia, María Victoria Martin, Leandra Lechner, Giselle M.A. Martínez-Noël, Graciela L. Salerno

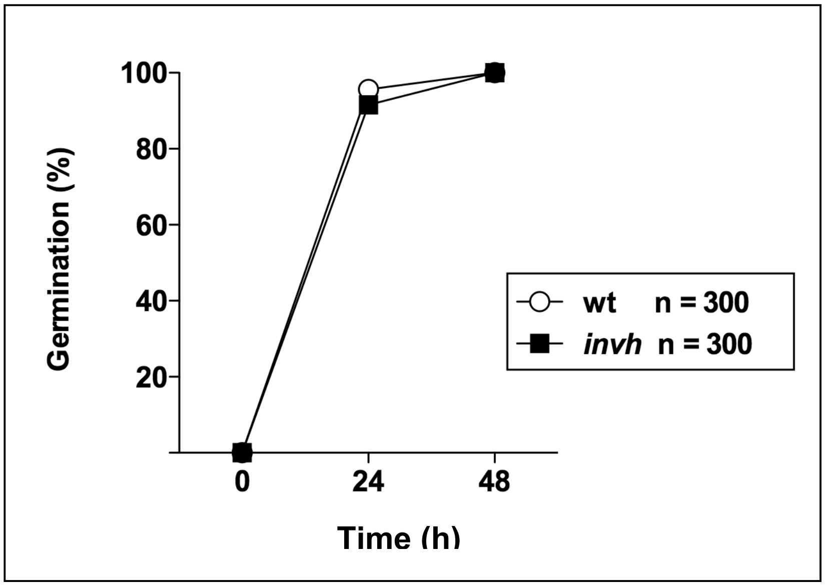

S4 Fig. Germination curve of Arabidopsis *invh* mutant and wt seeds. Seeds were surface-sterilized and plated on MS-1X containing 0.05% Mes-KOH (pH 5.7), 1% sucrose as carbon source and 0.8% agar. To break the dormancy, plates were maintained at 4ºC for 3 days in the dark prior to germination. Then seeds were incubated under controlled conditions of photoperiod and temperature (16 h/8 h, light/dark, 22±1ºC) and germination was registered under stereoscopic microscope to evaluate the visible radicle tip [34]. Three independent plates with one hundred wt or *invh* seeds were analyzed with. Average of 300 seeds ± SD.
