# Supplementary material for: The riddle of mitochondrial alkaline/neutral invertases: A novel Arabidopsis isoform mainly present in reproductive tissues and involved in root ROS production
Source: PLoS One. 2017 Sep 25;12(9):e0185286. doi: 10.1371/journal.pone.0185286 (PMC5612693; doi:10.1371/journal.pone.0185286)
Supplement: S5 Fig — Root length was evaluated on 7 days-old seedlings growing on vertical plates on MS medium (pH 5.7), solidified with 0.8% agar. To scale and quantify the root length, plates were photographed with a ruler on a side. Images were analyzed using ImageJ software. Statistical analysis was performed using Prism software. Data were collected from four biological replicates. Vertical bars represent the SE for n = 24. (PDF) [file pone.0185286.s007.pdf]

Supporting information

The riddle of mitochondrial alkaline/neutral invertases: A novel Arabidopsis isoform mainly present in reproductive tissues and involved in root ROS production.

Marina E. Battaglia, María Victoria Martin, Leandra Lechner, Giselle M.A. Martínez-Noël, Graciela L. Salerno

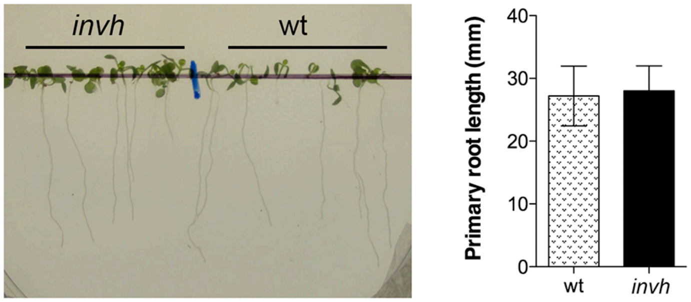

S5 Fig. Root length of *invh* mutant and wt Arabidopsis seedlings. Root length was evaluated on 7 days-old seedlings growing on vertical plates on MS medium (pH 5.7), solidified with 0.8% agar. To scale and quantify the root length, plates were photographed with a ruler on a side. Images were analyzed using ImageJ software. Statistical analysis was performed using Prism software. Data were collected from four biological replicates. Vertical bars represent the SE for n=24.
