# Supplementary material for: The riddle of mitochondrial alkaline/neutral invertases: A novel Arabidopsis isoform mainly present in reproductive tissues and involved in root ROS production
Source: PLoS One. 2017 Sep 25;12(9):e0185286. doi: 10.1371/journal.pone.0185286 (PMC5612693; doi:10.1371/journal.pone.0185286)
Supplement: S8 Fig — A/N-InvA (At1g56560, A/N-InvC (At3g06500), and A/N-InvH (At3g05820) expression analysis was performed from the GENEVESTIGATOR browser (www.genevestigator.com). Root category data were expanded. (PDF) [file pone.0185286.s010.pdf]

Supporting information

The riddle of mitochondrial alkaline/neutral invertases: A novel Arabidopsis isoform mainly present in reproductive tissues and involved in root ROS production.

Marina E. Battaglia, María Victoria Martin, Leandra Lechner, Giselle M.A. Martínez-Noël, Graciela L. Salerno

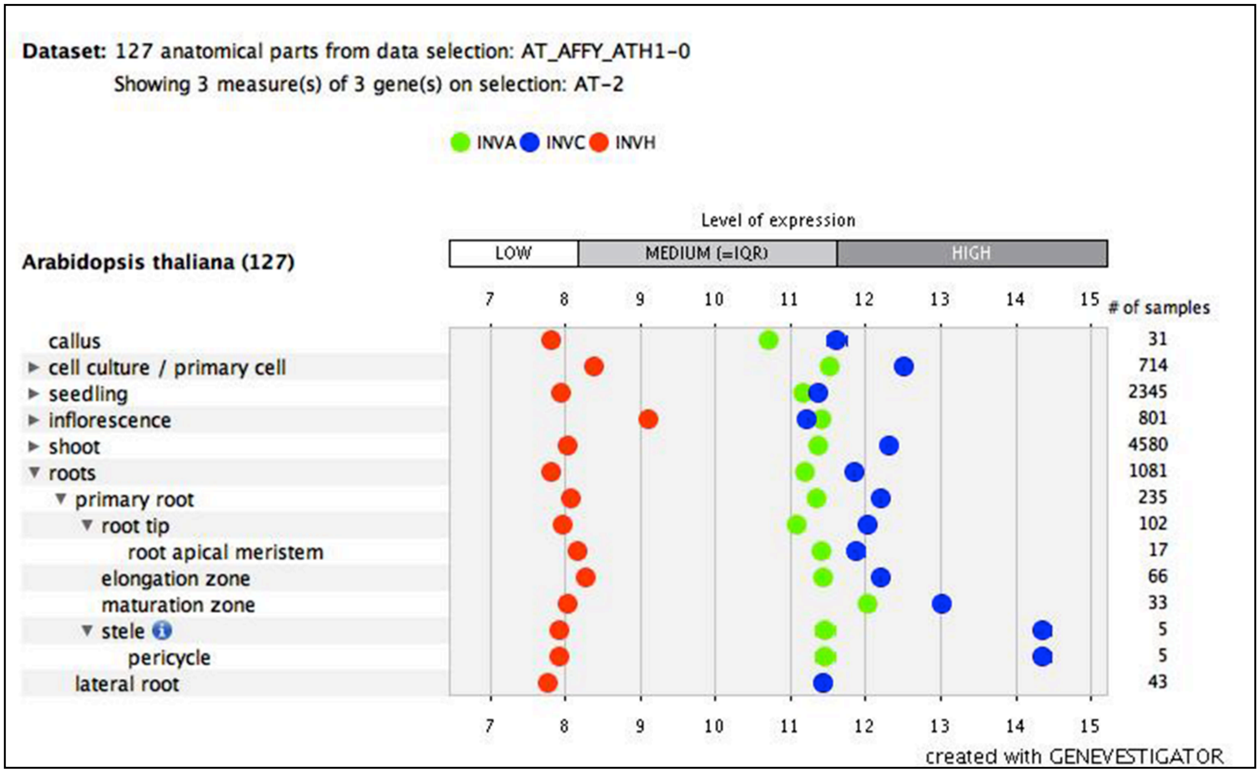

S8 Fig. Meta-profile analysis of *Arabidopsis thaliana* mitochondrial *A/N-Inv* gene expression. *A/N-InvA* (At1g56560, *A/N-InvC* (At3g06500), and *A/N-InvH* (At3g05820) expression analysis was performed from the GENEVESTIGATOR browser (www.genevestigator.com). Root category data were expanded.
