# Supplementary material for: The riddle of mitochondrial alkaline/neutral invertases: A novel Arabidopsis isoform mainly present in reproductive tissues and involved in root ROS production
Source: PLoS One. 2017 Sep 25;12(9):e0185286. doi: 10.1371/journal.pone.0185286 (PMC5612693; doi:10.1371/journal.pone.0185286)
Supplement: S9 Fig — GFP signals were quantified by confocal microscopy using ImageJ and correspond to images of A/N-InvH gene expression after salt (NaCl 100 mM), mannitol (200 mM), ABA (100 μM), and oxygen peroxide (1 mM) treatments. Seven days-old transgenic seedlings of wt plants expressing A/N-InvH promoter fused to GFP were exposed to 100 μM ABA, 100 mM NaCl or 200 mM mannitol for 24 h, on vertical plates with MS medium. Treatment with 1 mM H2O2 was performed for 30 min in MS solution. After treatments, GFP fluorescence was observed in a confocal microscope. All images were collected using the confocal parameters setting for control conditions. Control corresponds to non-transgenic wt plants to visualize autofluorescence. In all images equal areas were measured as described by in Martin et al. (2014) [46]. (PDF) [file pone.0185286.s011.pdf]

Supporting information

The riddle of mitochondrial alkaline/neutral invertases: A novel Arabidopsis isoform mainly present in reproductive tissues and involved in root ROS production.

Marina E. Battaglia, María Victoria Martin, Leandra Lechner, Giselle M.A. Martínez-Noël, Graciela L. Salerno

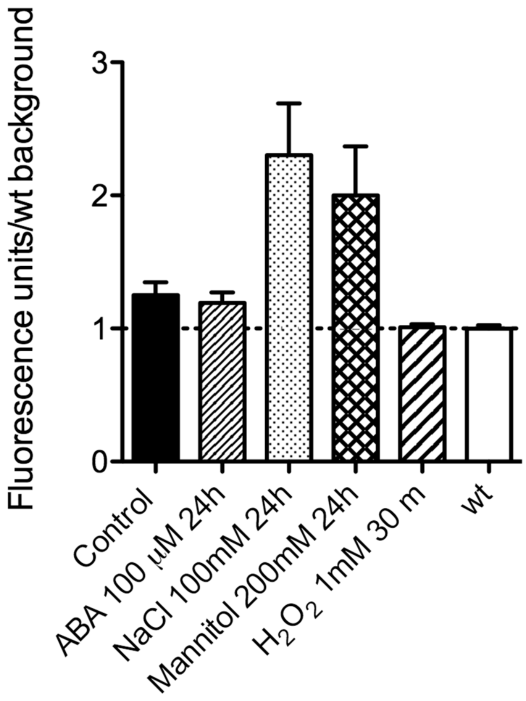

S9 Fig. Quantification of GFP fluorescence in root caps corresponding to Figure 7 images.

GFP signals were quantified by confocal microscopy using ImageJ and correspond to images of *A/N-InvH* gene expression after salt (NaCl 100 mM), mannitol (200 mM), ABA (100 μM), and oxygen peroxide (1 mM) treatments. Seven days-old transgenic seedlings of wt plants expressing *A/N-InvH* promoter fused to GFP were exposed to 100 μM ABA, 100 mM NaCl or 200 mM mannitol for 24 h, on vertical plates with MS medium. Treatment with 1 mM H_2O_2 was performed for 30 min in MS solution. After treatments, GFP fluorescence was observed in a confocal microscope. All images were collected using the confocal parameters setting for control conditions. Control corresponds to non-transgenic wt plants to visualize autofluorescence. In all images equal areas were measured as described by in Martin et al (2014) [46].
