# Supplementary material for: The riddle of mitochondrial alkaline/neutral invertases: A novel Arabidopsis isoform mainly present in reproductive tissues and involved in root ROS production
Source: PLoS One. 2017 Sep 25;12(9):e0185286. doi: 10.1371/journal.pone.0185286 (PMC5612693; doi:10.1371/journal.pone.0185286)
Supplement: S10 Fig — Expression data correspond to Arabidopsis primary stem (DataSet Record: GDS2895) from full-genome microarrays [Ehlting et al., Plant J 42:618–640 (2005)] deposited in GEO public data repository [Edgar et al., Nucleic Acids Res 30:207–210 (2002)]. (A-C) Expression of the three mitochondrial A/N-Inv genes [A/N-InvH (At3g05820), A/N-InvA (At1g56560) and A/N-InvC (At3g06500)] in two stages of development (5 cm, light green; 10 cm, green). Stems were cut in sections (from the apical meristem) indicated in the pink boxes. (PDF) [file pone.0185286.s012.pdf]

Supporting information

The riddle of mitochondrial alkaline/neutral invertases: A novel Arabidopsis isoform mainly present in reproductive tissues and involved in root ROS production.

Marina E. Battaglia, María Victoria Martin, Leandra Lechner, Giselle M.A. Martínez-Noël, Graciela L. Salerno

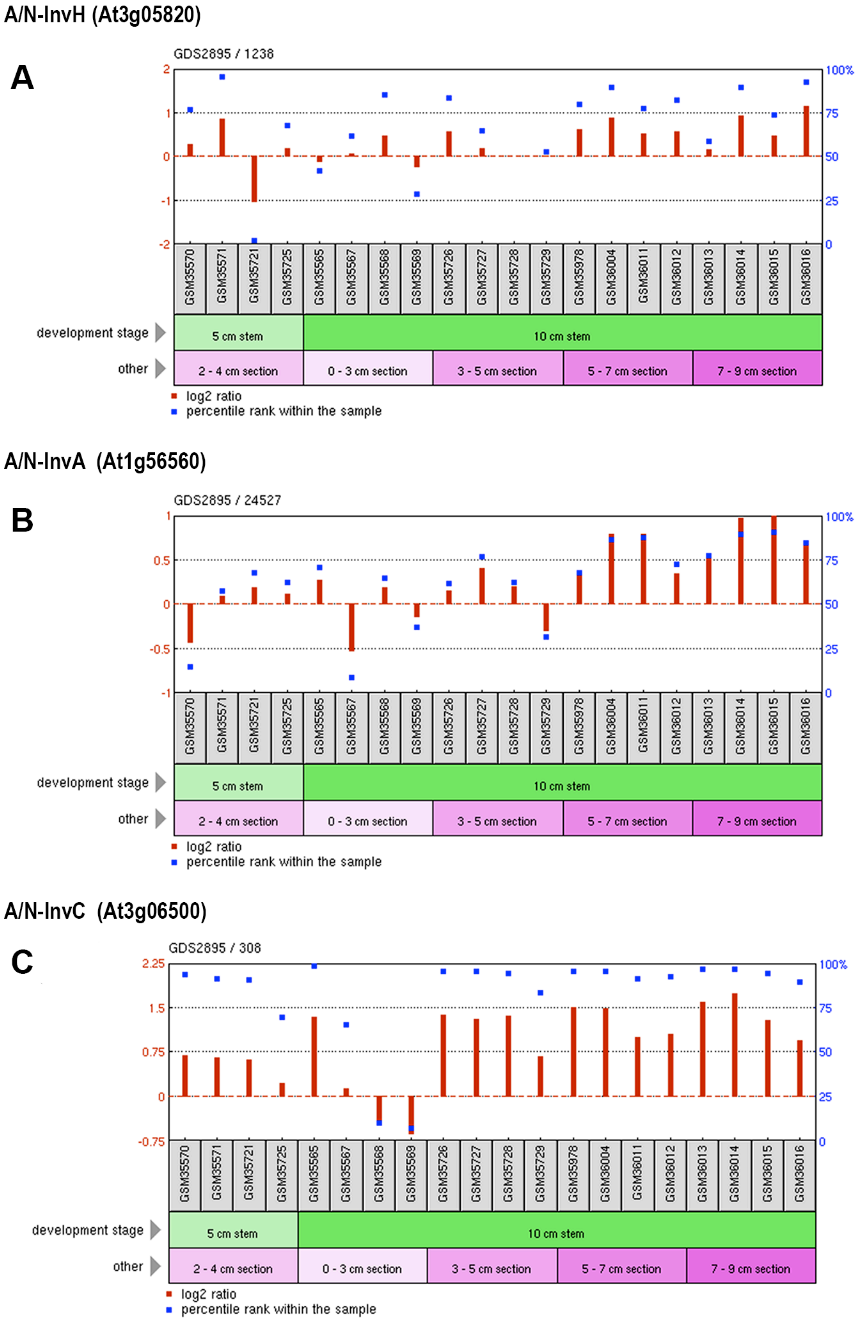

S10 Fig. Mitochondrial *A/N-Inv* expression in different segments of the primary stem of Arabidopsis plants. Expression data correspond to Arabidopsis primary stem (DataSet Record:

GDS2895) from full-genome microarrays [Ehlting et al., Plant J 42:618-640 (2005)] deposited in GEO public data repository [Edgar et al., Nucleic Acids Res 30:207-210 (2002)]. (A-C) Expression of the three mitochondrial *A/N-Inv* genes [*A/N-InvH* (At3g05820), *A/N-InvA* (At1g56560) and *A/N-InvC* (At3g06500)] in two stages of development (5 cm, light green; 10 cm, green). Stems were cut in sections (from the apical meristem) indicated in the pink boxes.
